# Financial incentives and coverage of child health interventions: a systematic review and meta-analysis

**DOI:** 10.1186/1471-2458-13-S3-S30

**Published:** 2013-09-17

**Authors:** Diego G Bassani, Paul Arora, Kerri Wazny, Michelle F Gaffey, Lindsey Lenters, Zulfiqar A Bhutta

**Affiliations:** 1 Centre for Global Child Health, The Hospital for Sick Children, Toronto, ON, Canada; 2 Dalla Lana School of Public Health, University of Toronto, Toronto, ON, Canada; 3 Department of Paediatrics, University of Toronto, Toronto, ON, Canada; 4 Division of Women and Child Health, Aga Khan University Hospital, Karachi, Pakistan

## Abstract

**Background:**

Financial incentives are widely used strategies to alleviate poverty, foster development, and improve health. Cash transfer programs, microcredit, user fee removal policies and voucher schemes that provide direct or indirect monetary incentives to households have been used for decades in Latin America, Sub-Saharan Africa, and more recently in Southeast Asia. Until now, no systematic review of the impact of financial incentives on coverage and uptake of health interventions targeting children under 5 years of age has been conducted. The objective of this review is to provide estimates on the effect of six types of financial incentive programs: (i) Unconditional cash transfers (CT), (ii) Conditional cash transfers (CCT), (iii) Microcredit (MC), (iv) Conditional Microcredit (CMC), (v) Voucher schemes (VS) and (vi) User fee removal (UFR) on the uptake and coverage of health interventions targeting children under the age of five years.

**Methods:**

We conducted systematic searches of a series of databases until September 1st, 2012, to identify relevant studies reporting on the impact of financial incentives on coverage of health interventions and behaviors targeting children under 5 years of age. The quality of the studies was assessed using the CHERG criteria. Meta-analyses were undertaken to estimate the effect when multiple studies meeting our inclusion criteria were available.

**Results:**

Our searches resulted in 1671 titles identified 25 studies reporting on the impact of financial incentive programs on 5 groups of coverage indicators: breastfeeding practices (breastfeeding incidence, proportion of children receiving colostrum and early initiation of breastfeeding, exclusive breastfeeding for six months and duration of breastfeeding); vaccination (coverage of full immunization, partial immunization and specific antigens); health care use (seeking healthcare when child was ill, visits to health facilities for preventive reasons, visits to health facilities for any reason, visits for health check-up including growth control); management of diarrhoeal disease (ORS use during diarrhea episode, continued feeding during diarrhea, healthcare during diarrhea episode) and other preventive health interventions (iron supplementation, vitamin A, zinc supplementation, preventive deworming). The quality of evidence on the effect of financial incentives on breastfeeding practices was low but seems to indicate a potential positive impact on receiving colostrum, early initiation of breastfeeding, exclusive breastfeeding and mean duration of exclusive breastfeeding. There is no effect of financial incentives on immunization coverage although there was moderate quality evidence of conditional cash transfers leading to a small but non-significant increase in coverage of age-appropriate immunization. There was low quality evidence of impact of CCT on healthcare use by children under age 5 (Risk difference: 0.14 [95%CI: 0.03; 0.26]) as well as low quality evidence of an effect of user fee removal on use of curative health services (RD=0.62 [0.41; 0.82]).

**Conclusions:**

Financial incentives may have potential to promote increased coverage of several important child health interventions, but the quality of evidence available is low. The more pronounced effects seem to be achieved by programs that directly removed user fees for access to health services. Some indication of effect were also observed for programs that conditioned financial incentives on participation in health education and attendance to health care visits. This finding suggest that the measured effect may be less a consequence of the financial incentive and more due to conditionalities addressing important informational barriers.

## Background

Financial incentives are becoming widely used policy strategies to alleviate poverty, foster several aspects of development, and improve the health of populations. It has also been recommended as an important strategy to reduce barriers to access to health care [1] and, more often than not, health gains are explicit objectives of these strategies [2]. Microcredit [3-5], user fee removal policies [6], voucher schemes [7] and cash transfer programs [8-11] that provide direct or indirect monetary incentives to households, with or without activity or behavioral conditionalities, have been used for decades in Latin American [9,12-14] and sub-Saharan African countries [15-19], and in Southeast Asian settings [20-24].

With an overarching goal of poverty alleviation, and an assumption that, in general, these policies will allow market mechanisms to help people overcome poverty, many complex and expensive programs have been implemented on a very large scale in some countries [7,10,25-27]. More often than not there is an expectation that care, uptake and coverage of health interventions, and ultimately health status, will improve as a consequence of such programs and policies [18,24,28-30], as the poorest sections of the population most often face the greatest barriers to accessing health services [2]. In most instances, these are financial barriers [1]; hence, removing such impediments should lead to an increase in the uptake of health interventions and care seeking in case of illness. Evaluations of large programs have shown a dose-response effect of the amount of money received on health status [14], suggesting it may act independently of the conditionality. In addition, many of these programs are conditional on school attendance [9,12,31], participating in health education activities [32-34], taking children to preventive health-care visits [9,25,31] and keeping vaccines up-to-date [7,10,12,13,25], which should improve health status. Some other programs offer health education activities [22,33,34] or streamline participants’ access to health care [26] in addition to the financial benefit offered, thereby addressing informational as well as financial barriers, but in many cases participation in such activities is not a condition for receiving the financial benefit.

Although previous systematic reviews and overviews [1,35-37] have addressed the impact of different types of financial incentive programs on health, no comprehensive systematic review has been conducted on the impact of a broad range of financial programs implemented in low- and middle-income countries on coverage and uptake of health interventions and behaviors targeting children under five years of age. The objective of this review is to provide estimates of the effect of six types of financial incentive programs on the uptake and coverage of such health interventions: (i) Unconditional cash transfers, (ii) Conditional cash transfers, (iii) Unconditional microcredit, (iv) Conditional microcredit, (v) Unconditional voucher (vi) Conditional voucher and (vi) User fee removal. These interventions are described in Table 1.

**Table 1:** Definitions of interventions included in the review

Intervention	Definition
Unconditional Cash Transfer	Monetary transfers to households or individuals without pre-imposed conditionalities.
Conditional Cash Transfer	Monetary transfers to households or individuals conditional on the recipient adopting and maintaining certain behaviors prescribed by the cash transfer program.
Unconditional Microcredit	Small loans offered to borrowers (usually lacking employment or credit history) without imposing conditionalities other than re-payment of the loaned amount.
Conditional Microcredit	Small loans offered to borrowers (usually lacking employment or credit history) conditional on the recipient adopting and maintaining certain behaviors prescribed by the program in addition to re-payment of the loaned amount.
Unconditional Voucher*	Indirect monetary transfer given by issue of coupons, vouchers, electronic card transfer or other method used to purchase commodities from local shops or outlets.
Conditional Voucher	Indirect monetary transfer given by issue of coupons, vouchers, electronic card transfer or other method used to purchase commodities from local shops or outlets conditional on the recipient adopting and maintaining certain behaviors prescribed by the voucher program.
User Fee Removal	Total or nearly total (75% or more) removal of user fees for accessing heath services.

* No unconditional voucher programs were included in this study.

## Methods

We systematically reviewed all studies published up to September 1st, 2012 to identify studies with data assessing the impact of financial incentives on access to child health interventions using the Child Health Evaluation Reference Group (CHERG) systematic review guidelines [38]. We conducted the initial search in March 2012 and updated searches on July 2012 and September 2012. The searches were completed using OvidSP to scan the Pubmed, EMBASE and AMED databases. We used all combinations of the following key search terms: Cash transfer, voucher scheme, demand side financing, social transfer, voucher program. We purposely included broad categories as well as names of financial schemes identified through previous reviews, other databases and repositories. We included in our search variations of names and/or acronyms of the thirty-five programs we identified in previous publications [1,2,17,18,28,35,39-41]. In addition, we searched variations of the terms microcredit, microfinance, micro-insurance, and economic empowerment, and limited the results of this search using variations of the terms evaluation or impact. To incorporate user fees, we adopted a previous review’s search strategy [42], and limited to children. Our search strategies are described in detail in Additional File 1.

We included randomized controlled trials (RCT), cluster randomized controlled trials (cRCT) and observational studies reported either in peer-reviewed journals or in institutional or commissioned reports that assessed the impact of financial incentive programs on health interventions targeting children under the age of five.

### Types of outcomes reported

Studies included in this review report on the impact of financial incentive programs on five groups of coverage indicators:

(a) Breastfeeding practices (breastfeeding incidence, feeding of colostrum, early initiation of breastfeeding, exclusive breastfeeding for 6 months and duration of breastfeeding);

(b) Vaccination (coverage of full vaccination, partial vaccination and specific vaccines);

(c) Health care use (preventive and curative health care use, visits to health facilities for preventive and curative reasons, visits to health facilities for check-up);

(d) Management of diarrheal diseases (ORS use, continued feeding and health care seeking);

(e) Other preventive health interventions (preventive deworming, vitamin A and iron supplementation).

A detailed description of the outcomes included in this review and the definitions used is presented in Table 2.

**Table 2:** Definitions of outcomes included in the review and effect measure reported

Outcome	Definition	Effect measure*
**Breastfeeding practices**		

Receiving colostrum	Percentage of newborns receiving colostrum	Mean difference in the change in percentage of newborns receiving colostrum between intervention and control group
Early initiation of breastfeeding	Percentage of newborns breastfed within the first hour of life	Mean difference in the change in percentage of early initiation of breastfeeding between intervention and control group
Exclusive breastfeeding	Percentage of infants 0 to 5 months who are exclusively breastfed	Mean difference in the change in percentage of exclusive breastfeeding between intervention and control group
Duration of exclusive breastfeeding	Mean duration of exclusive breasfeeding in days	Mean difference in the percent change in duration of exclusive breastfeeding between intervention and control group
Breastfeeding among children <2 years	Percentage of children under 2 years of age that are or were breastfed	Mean difference in the change in percentage of any breastfeeding between intervention and control group

**Vaccination**		

BCG coverage	Percentage of children that received BCG	Mean difference in the change in BCG coverage between intervention and control group
DPT-1 coverage	Percentage of children that received DPT-1 vaccine	Mean difference in the change in DPT-1 coverage between intervention and control group
DPT-3 coverage	Percentage of children that received DPT-3 vaccine	Mean difference in the change in DPT-3 coverage between intervention and control group
MCV coverage	Percentage of children that received measles (MCV) vaccine	Mean difference in the change in MCV coverage between intervention and control group
Polio vaccine coverage	Percentage of children that received polio vaccine	Mean difference in the change in OPV coverage between intervention and control group
Any vaccination coverage	Percentage of children that received any vaccine	Mean difference in the change in coverage of any antigen between intervention and control group
Full vaccination coverage	Percentage of children that are fully vaccinated according to the country's EPI schedule for their age	Mean difference in the change in coverage of EPI between intervention and control group

**Health care use**		

Preventive health care use	Percentage of children with a preventive health care visit in the previous 6 months**	Mean difference in the change in the percentage of children reporting a preventive health care visit between intervention and control group
Curative health care use	Percentage of children with a health care visit due to illness in the previous 6 months**	Mean difference in the change in the percentage of children reporting a curative health care visit between intervention and control group
Health care use	Percentage of children with any health care visit in the previous 6 months**	Mean difference in the change in the percentage of children reporting any health facility visit between intervention and control group
Preventive health care visits	Mean number of child-visits for preventive reasons in the previous month**	Mean difference in the percentage change in the number of preventive visits between intervention and control group
Curative health care visits	Mean number of child-visits due to illness in the previous month**	Mean difference in the percentage change in the number of curative visits between intervention and control group
New health care visits	Mean number of new child-visits in the previous month**	Mean difference in the percentage change in the number of new visits between intervention and control group
Follow-up health care visits	Mean number of follow-up child-visits after a curative visit in the previous month**	Mean difference in the percentage change in the number of follow-up visits between intervention and control group
Health care visits	Mean number of any child-visit in the previous month**	Mean difference in the percentage change in the number of any visits between intervention and control group

**Management of diarrhoeal disease**		

ORS use	Percentage of children that received oral rehydration solution during the last episode of diarrhoea	Mean difference in the change in percentage of ORS use during latest diarrhoea episode between intervention and control group
Continued feeding	Percentage of children that were fed the same amount or more than usual during the last episode of diarrhoea	Mean difference in the change in percentage of continued feeding during latest diarrhoea episode between intervention and control group
Care-seeking	Percentage of children that were taken to a health facility during the last episode of diarrhoea	Mean difference in the change in the percentage of children taken to health facility during latest diarrhoea episode between intervention and control group

**Other preventive health interventions**		

Preventive deworming	Percentage of children that received deworming drugs in the last 6 months**	Mean difference in the change in percentage of preventive deworming between intervention and control group
Vitamin A supplementation	Percentage of children that received Vitamin A supplementation in the last 6 months**	Mean difference in the change in percentage of vitamin A supplementation between intervention and control group
Iron supplementation	Percentage of children that received iron supplementation in the last 6 months**	Mean difference in the change in percentage of iron supplementation between intervention and control group

* In the case of cross-sectional studies, the effect measure was calculated assuming no change in control group and that the baseline value for the intervention group as equal to that of the control group.

** When reporting period is different this is noted as a limitation in the quality assessment table.

We abstracted all available data in duplicate for each of the outcomes and financial incentives described above We presented effect measures as mean risk differences-in-difference and their 95% confidence intervals. All analyses were done, using RevMan 5 (Cochrane Collaboration).

### Studies identified

**Figure 1:**
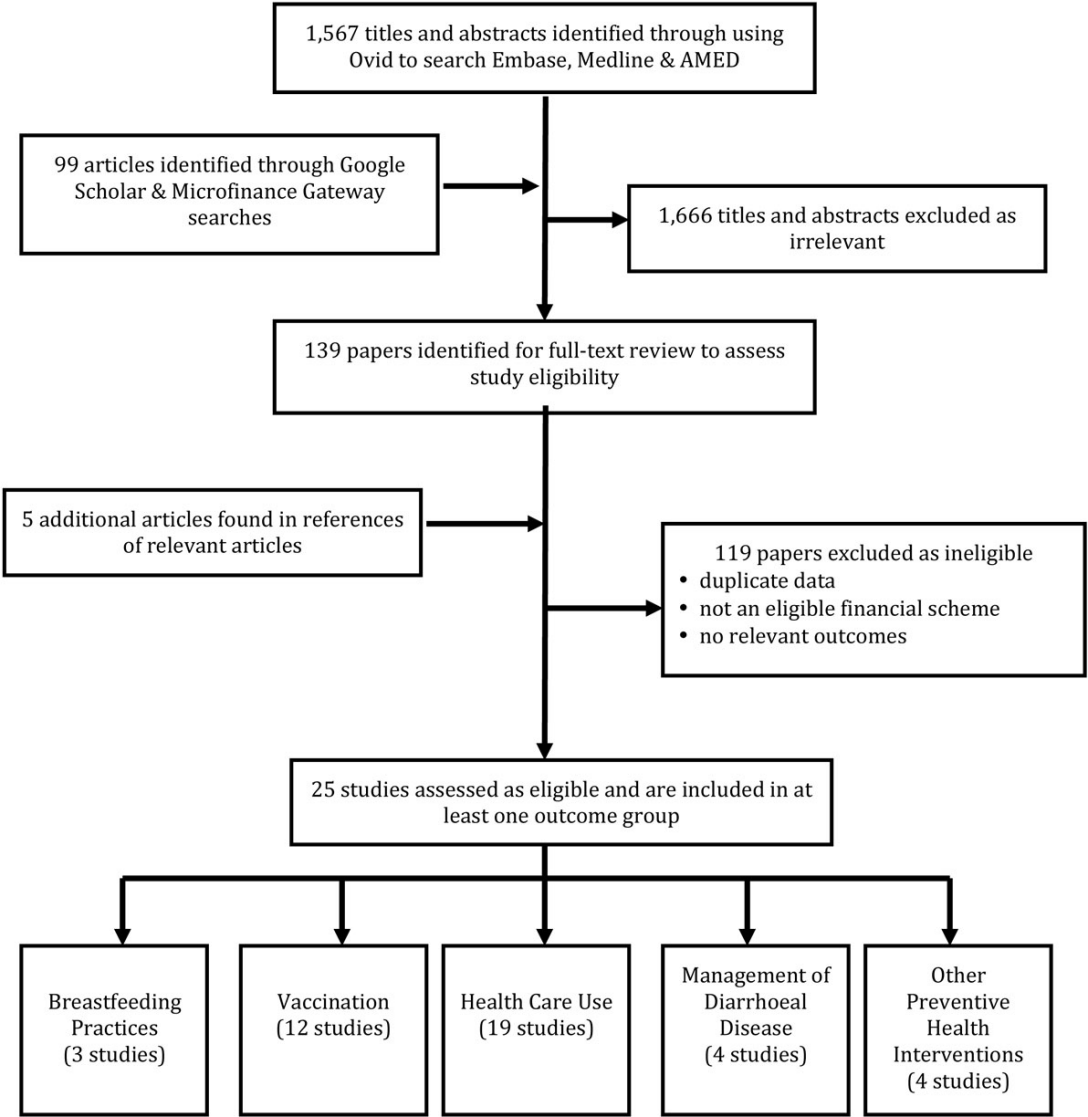
Flow diagram showing identification of included studies.

After removing duplicates, our searches yielded 1,567 titles. To ensure identification of all relevant literature, we scanned the references of all relevant articles identified through our searches. To complement our formal search strategy, we conducted a number of searches in Google Scholar. For these searches we used the names of identified conditional cash transfer, unconditional cash transfer, voucher-scheme, microfinance and food stamp programs combined with the terms evaluation and health and the country in which the program was carried out. Results were sorted by relevance and the titles and abstracts of the articles in the first ten pages of results were scanned for inclusion. In cases where titles and abstracts were not in English, titles and abstracts were translated using Google Translate. In cases where search results were obviously irrelevant, titles and abstracts were only scanned for the first five pages of results. Articles that had previously been found through the formal search strategy were not pulled again. A total of 78 Google Scholar searches were performed, in which 99 articles were identified as satisfying initial inclusion criteria. We also searched the Microfinance Gateway library and screened all publications relating to the terms health and nutrition. Of the 1,666 screened in duplicate based on titles and abstracts, 1,527 articles were excluded as obviously irrelevant. We thoroughly reviewed 139 full publications identified through our searches as well as an additional five articles that were located through scanning references of relevant articles, also in duplicate. We excluded 119 of these articles based on criteria defined a priori, either because they contained duplicate data to one of our included studies, did not include an eligible financial intervention, did not have a comparison group or relevant outcomes. In the end, 25 studies were included [7,8,10,15,16,21-23,25,26,33,34,40,42-53]. Figure 1 is a schematic representation of our search.

## Results

### Types of reports

Just under half the studies were institutional reports and thus were not peer-reviewed [8,10,25,33,34,43,45,47-50]. Only 36% of the studies were randomized trials. Of the 25 studies, 13 were in South America or the Caribbean [7,8,10,25,26,34,43-45,47-49,51], 8 were in Africa [15,16,33,40,42,50,52,53] and 4 were in South East Asia [21-23,46]. 48% of the studies evaluated cash transfer programs: 41% evaluated conditional cash transfer programs and 7% evaluated unconditional cash transfer programs. 22% of the programs evaluated the effects of removing user fees. One quarter of the studies evaluated microcredit programs. Almost half of the programs evaluated (48%) had a conditional component relating to health. For details of each study, see Additional file 2. We present forest plots only for selected outcomes. Additional file 3 presents forest plots for all study outcomes.

### Evidence of effect of financial incentives on breastfeeding practices

**Figure 2:**
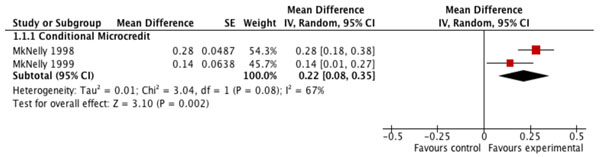
Effect of financial incentives on percentage of newborns receiving colostrum.

The overall quality of evidence for the effect of financial incentives on breastfeeding practices was low, mainly due to the limited number of relevant studies available (Table 3). The pooled estimate from two studies suggests that conditional microcredit programs produce an average 22% net increase in the percentage of newborns receiving colostrum (MD=0.22; CI: 0.08 to 0.35) compared to control (Figure 2). Evidence from another two microcredit studies suggests no statistically significant effect of either conditional (MD=-0.01; CI: -0.03 to 0.02) or unconditional (MD=-0.06; CI: -0.16 to 0.04) microcredit programs on the prevalence of any breastfeeding among children under two years (Table 3). Estimates of the effect of financial incentive programs on early initiation of breastfeeding and the prevalence and duration of exclusive breastfeeding (measured in months) among children under six months are based on single studies that were published as non-peer-reviewed reports. In four of the six studies included in these analyses of breastfeeding practices, the financial incentives were conditional on mothers’ participation in health and nutrition education sessions that included breastfeeding promotion.

**Table 3:** Quality assessment of effect estimates of financial incentives on coverage of breastfeeding practices

Intervention	No. of studies	Design	Limitations	Consistency	Generalizability to population of interest	Conditionalities related to outcome (no. of studies)	Overall quality of evidence	Mean difference (95% CI)
***Receiving colostrum***								

Conditional microcredit	2	Cluster RCT /Cohort	Analysis of cRCT does not account for clustering	Consistent and both studies show benefit	Bolivia and Ghana	Health and nutrition education (2)	Low	0.22 (0.08; 0.35)

***Early initiation of breastfeeding***								

Conditional microcredit	1	Cluster RCT	Single study. Analysis of cRCT does not account for clustering	-	Bolivia	Health and nutrition education (1)	Low	0.17 (0.01; 0.33)

***Exclusive breastfeeding***								

Conditional microcredit	1	Cluster RCT	Single study. Analysis of cRCT does not account for clustering	-	Bolivia	Health and nutrition education (1)	Low	0.20 (0.03; 0.37)

***Duration of exclusive breastfeeding***								

Conditional microcredit	1	Cohort	Only one study	-	Ghana	Health and nutrition education (1)	Low	11.49 (1.69; 21.29)

***Breastfeeding among children < 2 years***								

Unconditional microcredit	2	Cohort	Type of breastfeeding (e.g. exclusive, predominant) is not specified	Consistent, both studies show negative effect	Ecuador and Honduras	-	Low	-0.06 (-0.16; 0.04)
Conditional microcredit	2	Cohort	Type of breastfeeding (e.g. exclusive, predominant) is not specified	Inconsistent	Ecuador and Honduras	Health and nutrition education (2)	Low	-0.01 (-0.03; 0.02)

### Evidence of effect of financial incentives on immunization

**Figure 3:**
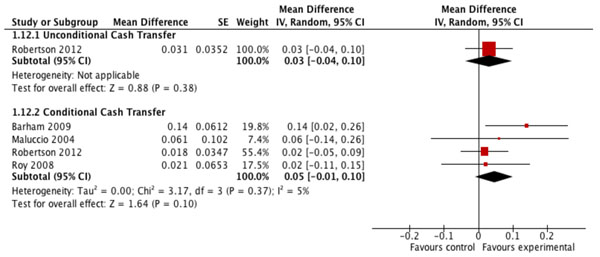
Effect of financial incentives on percentage of children receiving their full EPI vaccine schedule.

There is moderate or low quality evidence from conditional cash transfer and conditional microcredit programs indicating no significant effect of either of these types of financial incentive on the coverage of BCG, DPT-1, DPT-3, measles or polio vaccination or on the coverage of any vaccination (Table 4). Financial incentives in many of the studies included in these analyses were conditional on children attending preventive healthcare visits that included vaccination (Table 4). However, moderate quality evidence compiled from four studies suggests that conditional transfer programs may increase coverage of full, age-appropriate vaccination (MD=0.05; CI: -0.01 to 0.10), but this pooled estimate is not statistically significant (Figure 3). Estimates of the vaccination coverage effects of unconditional cash transfer or unconditional microcredit programs, or of conditional voucher schemes, are based only on single studies, some of which were published as non-peer-reviewed reports.

**Table 4:** Quality assessment of effect estimates of financial incentives on coverage of child vaccination

Intervention	No. of studies	Design	Limitations	Consistency	Generalizability to population of interest	Conditionalities related to outcome (no. of studies)	Overall quality of evidence	Mean difference (95% CI)
*** BCG coverage***								

Conditional cash transfer	3	RCT/Cluster RCT/Cohort	>20% attrition in cohort study and not peer-reviewed	Inconsistent	Bangladesh, Jamaica and Nicaragua	Preventive health visits (2)	Moderate	0.00 (-0.04; 0.04)
Conditional microcredit	2	Cluster RCT/Cohort	Analysis of cRCT does not account for clustering.	Consistent, both studies show benefit	Bolivia and Ghana	Preventive health visits (1)	Low	0.09 (-0.02; 0.20)

***DPT-1 coverage***								

Conditional cash transfer	2	RCT/Cross-sectional	Reverse causality possible in one study which is also not peer-reviewed	Inconsistent	Bangladesh and Colombia	Preventive health visits (1)	Low	0.06 (-0.01; 0.12)
Unconditional microcredit	1	Cross-sectional	Only one study	-	Bangladesh	-	Low	-0.02 (-0.19; 0.15)
Conditional microcredit	2	Cluster RCT/Cohort	Analysis of cRCT does not account for clustering.	Inconsistent	Bolivia and Ghana	Health education (2)	Low	-0.02 (-0.23; 0.19)
Conditional voucher	1	Cluster RCT	Only one study	-	Honduras	Preventive health visits (1)	Low	0.07 (0.01; 0.13)

***DPT-3 coverage***								

Conditional cash transfer	3	RCT/Cluster RCT/Cohort	>20% attrition in cohort study and not peer-reviewed	Inconsistent	Bangladesh, Jamaica and Nicaragua	Preventive health visits (2)	Moderate	0.01 (-0.03; 0.06)
Conditional microcredit	2	Cluster RCT/Cohort	Analysis of cRCT does not account for clustering.	Inconsistent	Bolivia and Ghana	Health education (2)	Low	0.03 (-0.20; 0.27)

***MVC coverage***								

Conditional cash transfer	3	RCT/Cluster RCT/Cohort	>20% attrition in cohort study and not peer-reviewed	Inconsistent	Bangladesh, Jamaica and Nicaragua	Preventive health visits (2)	Moderate	-0.01 (-0.11; 0.09)
Unconditional microcredit	1	Cross-sectional	Only one study	-	Bangladesh	-	Low	0.09 (0.08; 0.11)
Conditional microcredit	2	Cluster RCT/Cohort	Analysis of cRCT does not account for clustering	Inconsistent	Bolivia and Ghana	Health education (2)	Low	-0.04 (-0.46; 0.38)
Conditional voucher	1	Cluster RCT	Only one study	-	Honduras	Preventive health visits (1)	Low	0.00 (-0.09; 0.09)

***OPV-3 coverage***								

Conditional cash transfer	3	RCT/Cluster RCT /Cohort	>20% attrition in cohort study and not peer-reviewed	Inconsistent	Bangladesh, Jamaica and Nicaragua	Preventive health visits (2)	Moderate	0.03 (-0.04; 0.11)
Conditional microcredit	2	Cluster RCT/Cohort	Analysis of cRCT does not account for clustering	Consistent, both studies show negative effect	Bolivia and Ghana	Health education (2)	Low	-0.07 (-0.18; 0.03)

***Any vaccination coverage***								

Conditional cash transfer	1	Cross-sectional	Only one study	-	Peru	Preventive health visits (1)	Low	0.22 (0.12; 0.32)
Unconditional microcredit	1	Cross-sectional	Only one study	-	Pakistan	-	Low	0.08 (-0.00; 0.17)
Conditional microcredit	2	Cluster RCT/Cohort	Analysis of cRCT does not account for clustering	Inconsistent	Bolivia and Ghana	Health education (2)	Low	0.06 (-0.21; 0.34)

***Full vaccination coverage***								

Unconditional cash transfer	1	Cluster RCT	Study not published yet	-	Zimbabwe	-	Low	0.03 (-0.04; 0.10)
Conditional cash transfer	4	RCT/Cluster RCT	Different age groups (<2y and <5y)	Consistent, all studies show benefit	Nicaragua, Bangladesh, Zimbabwe	Immunization and preventive health visits (3)	Moderate	0.05 (-0.01; 0.10)

### Evidence of effect of financial incentives on health care use

**Figure 4:**
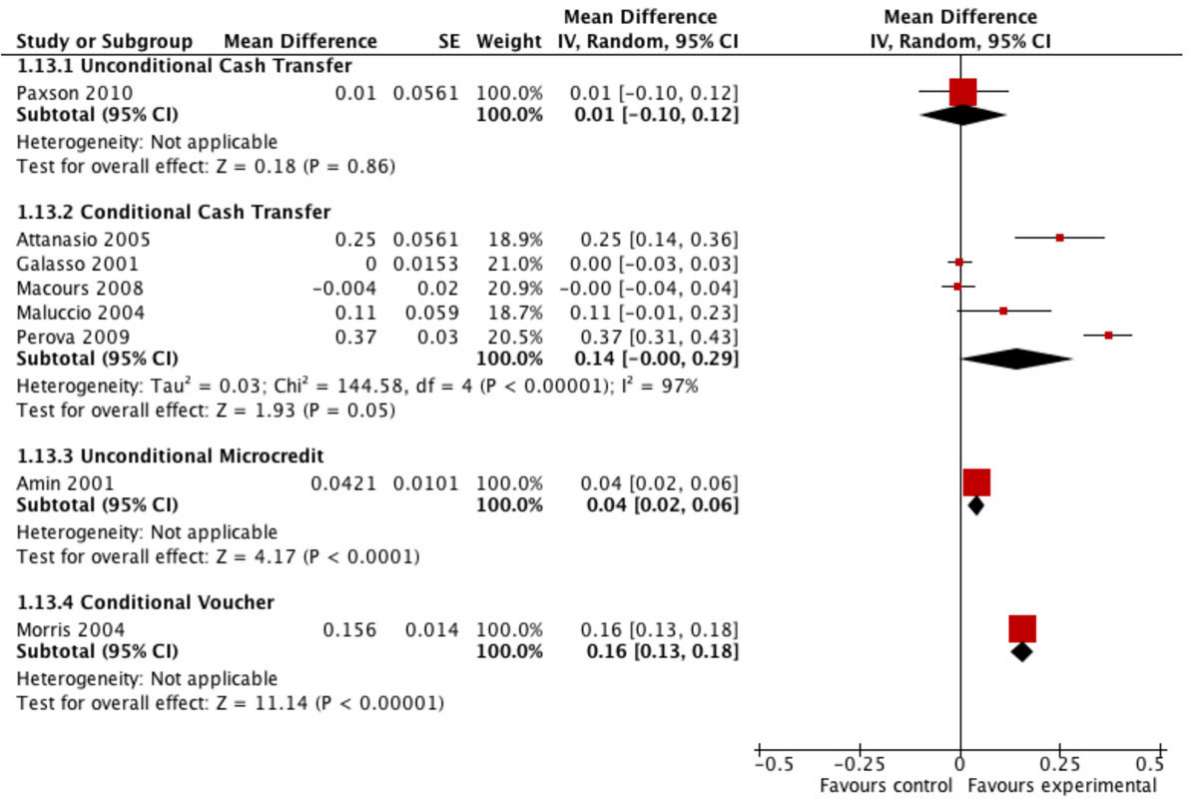
Effect of financial incentives on percentage of children accessing preventive health care in the previous 6 months.

The pooled analysis of five studies evaluating the impact of conditional cash transfer programs on the prevalence of preventive health care use by children shows an average 14% net increase among program participants compared to non-participants (MD=0.14; CI: -0.00 to 0.29) (Figure 4). The evidence is inconsistent across studies however, even though the financial incentives in four of the five programs were conditional on preventive health visit attendance, and the overall quality of this evidence is low given the variability in study designs, and because only one study was reported in a peer-reviewed publication (Table 5). Even more pronounced effects were observed for user fee removal on the prevalence (MD=0.33; CI: 0.24 to 0.43) and on the frequency (MD=0.99; CI: 0.71 to 1.27) (Table 5) of curative health care use, but the overall quality of the evidence for these effects was also low, with the pooled estimates based on only two studies each, none of which were randomized. Large and statistically significant effects of user fee removal on the frequency of other types of child health care visits were also shown in several individual studies, but these single study estimates yield low quality evidence only.

**Table 5:** Quality assessment of effect estimates of financial incentives on coverage of child health care use

Intervention	No. of studies	Design	Limitations	Consistency	Generalizability to population of interest	Conditionalities related to outcome (no. of studies)	Overall quality of evidence	Mean difference (95% CI)
***Preventive health care use***								

Unconditional cash transfer	1	Cluster RCT	Only one study	-	Ecuador	Preventive health visits, but conditionality was not implemented (1)	Low	0.01 (-0.10; 0.12)
Conditional cash transfer	5	Cluster RCT/Cohort/ Longitudinal panel/Cross-sectional	Variability in study design, reporting periods and only one peer-reviewed study	Inconsistent	Chile, Colombia, Nicaragua, Peru	Preventive health visits (4)	Low	0.14 (-0.00; 0.29)
Unconditional microcredit	1	Cross-sectional	Only one study	-	Bangladesh	-	Low	0.04 (0.02; 0.06)
Conditional voucher	1	Cluster RCT	Only one study and shorter reporting period	-	Honduras	Preventive health visits (1)	Low	0.16 (0.13; 0.18)

***Curative health care use***								

Conditional cash transfer	1	Cross-sectional	Only one study	-	Peru	Preventive health visits (1)	Low	0.22 (0.12; 0.32)
Unconditional microcredit	2	Cross-sectional	Reverse causality possible in all studies	Consistent	Bangladesh and Pakistan	-	Low	0.10 (0.07; 0.13)
User fee removal	2	Cross-sectional /Before and after design using administrative data	Individual-level data in one study and clinic-level data in the other, neither experimental	Consistent, both studies show benefit	Rwanda, Sudan	-	Low	0.33 (0.24; 0.43)

***Health care use***								

Conditional cash transfer	1	Longitudinal panel	Only one study	-	Brazil	Preventive health visits (1)	Low	0.04 (-0.02; 0.10)

***Preventive health care visits***								

Conditional cash transfer	1	Cohort	Only one study	-	Jamaica	Preventive health visits (1)	Low	0.38 (0.15; 0.62)
User fee removal	1	Before and after design using administrative data	Only one study	-	South Africa	-	Low	-0.03 (-0.18; 0.13)

***Curative health care visits***								

User fee removal	2	Before and after design using administrative data	No control group, one study limits the outcome to visits due to malaria only	Consistent, both studies show benefit	Niger and Kenya	-	Low	0.99 (0.71; 1.27)

***New health care visits***								

User fee removal	1	Before and after design using administrative data	Only one study	-	Uganda	-	Low	0.27 (0.18; 0.37)

***Follow-up health care visits***								

User fee removal	1	Before and after design using administrative data	Only one study	-	Uganda	-	Low	0.81 (0.73; 0.90)

***Health care visits***								

Conditional cash transfer	1	Cluster RCT	Only one study	-	Mexico	Preventive health visits (1)	Low	-0.01 (-0.02; -0.00)
User fee removal	1	Before and after design using administrative data	Clinic-level data	-	Uganda	-	Low	0.20 (0.10; 0.29)

### Evidence of effect of financial incentives on diarrhoea management

The overall quality of evidence for the effect of financial incentive programs on diarrhoea management outcomes was low, with only single studies of unconditional or conditional microcredit programs reporting on the use of oral rehydration solutions or on care-seeking during diarrhoea (Table 6). Two studies of conditional microcredit programs reported on the practice of continuing child feeding during diarrhoea, with the pooled estimate suggesting no effect of this type of financial incentive on this outcome (Table 6) despite the conditionality of mothers’ attendance of health and nutrition education sessions to qualify for microcredit in both studies.

**Table 6:** Quality assessment of effect estimates of financial incentives on management of diarrhoeal disease

Intervention	No. of studies	Design	Limitations	Consistency	Generalizability to population of interest	Conditionalities related to outcome (no. of studies)	Overall quality of evidence	Mean difference (95% CI)
***ORS use***								

Unconditional microcredit	1	Cross-sectional	Only one study	-	Pakistan	-	Low	0.02 (-0.02; 0.05)
Conditional microcredit	1	Cohort	Only one study	-	Ghana	Health and nutrition education (1)	Low	0.65 (0.53; 0.77)

***Continued feeding***								

Conditional microcredit	2	Cluster RCT/Cohort	Only two studies; analysis of cRCT does not account for clustering	Consistent	Bolivia and Ghana	Health and nutrition education (2)	Low	0.03 (-0.07; 0.13)

***Care-seeking***								

Conditional cash transfer	1	Cluster RCT	Only one study; outcome not limited to diarrhea, includes consultations for other diseases	-	Nicaragua	Children’s health service attendance, but not monitored (1)	Low	0.03 (-0.03; 0.09)

### Evidence of effect of financial incentives on coverage of other preventive health practices

All available evidence for the effects of financial incentives on other preventive health practices come from randomized or cluster randomized studies of unconditional or conditional cash transfer programs (Table 7). However, only single studies report on deworming and iron supplementation, yielding low quality evidence for these outcomes. Moderate quality evidence pooled from two randomized studies suggests that conditional cash transfer programs may increase the coverage of vitamin A supplementation (MD=0.16; CI: -0.01 to 0.34), but this pooled effect estimate is not statistically significant (Table 7). The conditionality attached to only one of the two conditional cash transfer programs was health-related, but this conditionality was not monitored.

**Table 7:** Quality assessment of effect estimates of financial incentives on coverage of other preventive health interventions

Intervention	No. of studies	Design	Limitations	Consistency	Generalizability to population of interest	Conditionalities related to outcome (no. of studies)	Overall quality of evidence	Mean difference (95% CI)
***Preventive deworming***								

Unconditional cash transfer	1	Cluster RCT	Only one study	-	Ecuador*	Preventive health visits, but conditionality was not implemented (1)	Low	0.08 (0.01; 0.15)
Conditional cash transfer	1	Cluster RCT	Only one study	-	Nicaragua**	Preventive health visits, but condition was not monitored (1)	Low	0.08 (0.00; 0.16)

***Vitamin A supplemention***								

Unconditional cash transfer	1	Cluster RCT	Only one study	-	Ecuador*	Preventive health visits, but conditionality was not implemented (1)	Low	0.01 (-0.03; 0.04)
Conditional cash transfer	2	RCT /Cluster RCT	Study in Nicaragua included three different CCT interventions but all were analyzed together	Consistent, both studies show benefit	Bangladesh* and Nicaragua**	Preventive health visits, but condition was not monitored (1)	Moderate	0.16 (-0.01; 0.34)

***Iron supplementation***								

Unconditional cash transfer	1	Cluster RCT	Only one study	-	Ecuador*	Preventive health visits, but conditionality was not implemented (1)	Low	0.01 (-0.03; 0.05)
Conditional cash transfer	1	Cluster RCT	Only one study	-	Nicaragua**	Children’s health service attendance (1)	Low	0.36 (0.25; 0.47)

* 12-month reporting period, ** 4-month reporting period

## Discussion

The apparent appeal of financial incentives is based in part on the underlying assumption that these programs will impact child health. Because there were indications of impacts on some child health outcomes [18], we hypothesized that improved access to health care and increases in coverage of child health interventions must be important components of the pathway from the implementation of financial incentive programs to child health gains. However, our main finding is that there is no high or moderate quality evidence to support this hypothesis. Our results reveal that the evidence for an impact of financial incentive programs on the coverage of a broad range of health interventions among children under five years is generally limited and of low quality. Although evidence on a few specific outcomes may be at maximum moderate, there is only low quality evidence of an effect of financial incentives on the groups of outcomes studied: breastfeeding practices, preventive deworming, health care use in case of illness and preventative health care use.

Reduction or elimination of user fees is one of the few interventions that had very large effects in the use of health services. Although the quality of the evidence is also low, the pronounced effects that were observed for user fee removal on health care use deserves attention. Nevertheless, it should be noted that one study observed a negative effect of generalized user fee removal policies on service use by children and pregnant women [15]. Such effect may be explained by difficulties of the health services in meeting increased demand, and further research is needed to clarify this association.

The role of conditionalities is one of the most important aspects to be addressed when evaluating the impact of financial incentive programs on health. Even in the limited number of studies in our review, it appears that conditioning financial incentives on health-related behaviors significantly influences program effect. It is challenging to attribute the health effects of conditional financial incentive programs to the monetary component because, theoretically, conditionality may be confounding this effect and also because programs are not designed to allow its evaluations to separate the effects.

Among the studies included in our review, in most cases the conditionality is related to participation in health activities that are directly related to the health outcome of interest. It has been previously noted that these health education or knowledge-transfer activities do increase coverage of interventions [1], therefore it is not surprising to notice that in our results all the positive effects observed for the group of breastfeeding outcomes, for example, come from programs that were conditional on women’s participation in health and nutritional education activities, all of which had a strong emphasis on breastfeeding promotion (Table 3). Similarly, the effect of conditional transfer programs on the coverage of full, age-appropriate vaccination, even though not statistically significant, is based on the pooled results of four studies, three of which were conditional on the participant maintaining vaccines up to date.

To strengthen this point, four of the five studies evaluating the impact of conditional cash transfer programs on preventive health care use were conditional on children attending preventive health care services routinely. Under such circumstances, it may be surprising that the pooled analysis yielded only a moderate 14% net increase among program participants. This difficulty in interpreting results of conditional financial incentives has been noted in a previous discussion about financial incentive programs [2], and indeed, isolating the effects of financial and non-financial program components is a daunting but necessary task that should be incorporated in the design of future evaluations of such programs.

The quantitative evidence for an effect of financial incentives and policies on the coverage of child health interventions presented here does not support the positive findings of earlier qualitative assessments of such programs [18,35]. Because the evidence is currently limited and of low quality, we plan to conduct systematic updates of this analysis as new studies and evaluations of such interventions become available. In addition, a similar exercise to systematically evaluate the evidence of the impact of such programs on other aspects of child health and development, such as morbidity and mortality, is warranted.

## Competing interests

The authors declare that they have no competing interests.

## Authors’ contributions

ZAB conceived of the study; DGB, PA, KW and ZAB wrote the protocol; KW conducted the literature searches, and abstracted the data with PA and DGB. KW and LL assessed study quality, with DGB and MFG resolving discrepancies; DGB and MFG analyzed the data and wrote the first draft of the manuscript; all authors revised the draft and approved the final manuscript.

## Supplementary Material

Additional File 1 Electronic search strategy for MEDLINE, EMBASE and AMED databases. Click here for file

Additional File 2 Abstracted data from all 25 studies included in the quantitative data synthesis. Click here for file

Additional File 3 Forest plots for all outcomes. Click here for file

## Declaration

The publication costs for this supplement were funded by a grant from the Bill & Melinda Gates Foundation to the US Fund for UNICEF (grant 43386 to "Promote evidence-based decision making in designing maternal, neonatal, and child health interventions in low- and middle-income countries”). The Supplement Editor is the principal investigator and lead in the development of the Lives Saved Tool (LiST), supported by grant 43386. He declares that he has no competing interests.

This article has been published as part of *BMC Public Health* Volume 13 Supplement 3, 2013: The Lives Saved Tool in 2013: new capabilities and applications. The full contents of the supplement are available online at http://www.biomedcentral.com/bmcpublichealth/supplements/13/S3.

## References

[B1] Chopra M Sharkey A Dalmiya N Anthony D Binkin N Strategies to improve health coverage and narrow the equity gap in child survival, health, and nutrition Lancet 2012 380 9850 1331 1340 10.1016/S0140-6736(12)61423-8 22999430

[B2] World Health Organization Public health agencies and cash transfer programmes : making the case for greater involvement 2011 Geneva: World Health Organization

[B3] Hamad R Fernald LC Microcredit participation and nutrition outcomes among women in Peru J Epidemiol Community Health 2012 66 6 e1 10.1136/jech.2010.108399 21051776

[B4] Norwood C Women, microcredit and family planning practices: a case study from rural Ghana J Asian Afr Stud 2011 46 2 169 183 10.1177/0021909610388747 21901899

[B5] Gautam Hazarika B G-K Household Access to Microcredit and Children’s Food Security in Rural Malawi: A Gender Perspective 2008 1 18

[B6] Amouzou A Habi O Bensaid K Reduction in child mortality in Niger: a Countdown to 2015 country case study Lancet 2012 380 9848 1169 1178 10.1016/S0140-6736(12)61376-2 22999428

[B7] Morris SS Flores R Olinto P Medina JM Monetary incentives in primary health care and effects on use and coverage of preventive health care interventions in rural Honduras: cluster randomised trial Lancet 2004 364 9450 2030 2037 10.1016/S0140-6736(04)17515-6 15582060

[B8] Paxson C Schady N Does Money Matter? The Effects of Cash Transfers on Child Development in Rural Ecuador Economic Development and Cultural Change 2010 59 1 187 229 10.1086/655458 20821896

[B9] Paes-Sousa R Santos LM Miazaki ES Effects of a conditional cash transfer programme on child nutrition in Brazil Bulletin of the World Health Organization 2011 89 7 496 503 10.2471/BLT.10.084202 21734763 PMC3127265

[B10] Maluccio J Flores R Impact Evaluation of a Conditional Cash Transfer Program: The Nicaraguan Red de Protección Social”, Fund Discussion Paper 2004 184

[B11] Aguero J Carter MR Woolard I The impact of unconditional cash transfers on nutrition: The South African Child Support Grant 2007

[B12] Fernald LC Hidrobo M Effect of Ecuador's cash transfer program (Bono de Desarrollo Humano) on child development in infants and toddlers: a randomized effectiveness trial Soc Sci Med 2011 72 9 1437 1446 21531060 10.1016/j.socscimed.2011.03.005

[B13] Attanasio O Meghir C Schady N Mexico's conditional cash transfer programme Lancet 2010 375 9719 980 981 author reply 10.1016/S0140-6736(10)60432-1 20304236

[B14] Fernald LC Gertler PJ Neufeld LM 10-year effect of Oportunidades, Mexico's conditional cash transfer programme, on child growth, cognition, language, and behaviour: a longitudinal follow-up study Lancet 2009 374 9706 1997 2005 10.1016/S0140-6736(09)61676-7 19892392

[B15] Wilkinson D Gouws E Sach M Karim SS Effect of removing user fees on attendance for curative and preventive primary health care services in rural South Africa Bulletin of the World Health Organization 2001 79 7 665 671 11477970 PMC2566476

[B16] Burgert CR Bigogo G Adazu K Odhiambo F Buehler J Breiman RF Laserson K Hamel MJ Feikin DR Impact of implementation of free high-quality health care on health facility attendance by sick children in rural western Kenya Trop Med Int Health 2011 16 6 711 720 10.1111/j.1365-3156.2011.02752.x 21447057

[B17] Palmer N Mueller DH Gilson L Mills A Haines A Health financing to promote access in low income settings-how much do we know? Lancet 2004 364 9442 1365 1370 10.1016/S0140-6736(04)17195-X 15474141

[B18] Lagarde M Haines A Palmer N Conditional cash transfers for improving uptake of health interventions in low- and middle-income countries: a systematic review JAMA 2007 298 16 1900 1910 10.1001/jama.298.16.1900 17954541

[B19] Bryant JH Kenya's cash transfer program: protecting the health and human rights of orphans and vulnerable children Health Hum Rights 2009 11 2 65 76 20845842

[B20] Ahmed AU Shams Y Nutritional effects of cash versus commodity-based public works programs. Bangladesh Food Policy Project Manuscript 63. Washington, DC: International Food Policy Research Institute International Food Policy Research Institute 1994

[B21] Amin R St Pierre M Ahmed A Haq R Integration of an essential services package (ESP) in child and reproductive health and family planning with a micro-credit program for poor women: experience from a pilot project in rural Bangladesh World Development 2001 29 9 1611 1621 10.1016/S0305-750X(01)00055-9

[B22] Roy SK Bilkes F Islam K Ara G Tanner P Wosk I Rahman AS Chakraborty B Jolly SP Khatun W Impact of pilot project of Rural Maintenance Programme (RMP) on destitute women: CARE, Bangladesh Food and nutrition bulletin 2008 29 1 67 75 18510207 10.1177/156482650802900108

[B23] Montgomery H Weiss J Can Commercially-oriented Microfinance Help Meet the Millennium Development Goals? Evidence from Pakistan World Development 2011 39 1 87 109 10.1016/j.worlddev.2010.09.001

[B24] Gopalan SS Durairaj V Addressing maternal healthcare through demand side financial incentives: experience of Janani Suraksha Yojana program in India BMC Health Serv Res 2012 12 319 10.1186/1472-6963-12-319 22978630 PMC3470975

[B25] Saude Md Avaliacao do Programa Bolsa-Alimentacao 2005 Brasilia: Government of Brazil 1 118

[B26] Galasso E Alleviating extreme poverty in Chile: the short term effects of Chile Solidario Estudios de Economía 2001 38 1 102

[B27] Wilkinson D Sach ME Abdool Karim SS Examination of attendance patterns before and after introduction of South Africa's policy of free health care for children aged under 6 years and pregnant women Bmj 1997 314 7085 940 941 10.1136/bmj.314.7085.940 9099118 PMC2126388

[B28] Cash transfers for children--investing into the future Lancet 2009 373 9682 2172 19560581 10.1016/S0140-6736(09)61166-1

[B29] Oliver A Brown LD A consideration of user financial incentives to address health inequalities J Health Polit Policy Law 2012 37 2 201 226 10.1215/03616878-1538602 22147947

[B30] Oliver A Can financial incentives improve health equity? Bmj 2009 339 b3847 10.1136/bmj.b3847 19778977

[B31] Fernald LC Gertler PJ Neufeld LM Role of cash in conditional cash transfer programmes for child health, growth, and development: an analysis of Mexico's Oportunidades Lancet 2008 371 9615 828 837 10.1016/S0140-6736(08)60382-7 18328930 PMC2779574

[B32] Barber SL Gertler PJ Empowering women to obtain high quality care: evidence from an evaluation of Mexico's conditional cash transfer programme Health Policy Plan 2009 24 1 18 25 19022854 10.1093/heapol/czn039 PMC2724849

[B33] MkNelly B Dunford C Impact of credit with education on mothers and their young children’s nutrition: Lower Pra Rural Bank credit with education program in Ghana Freedom from Hunger Research Paper 1998

[B34] MkNelly B Dunford C Impact of credit with education on mothers and their young children’s nutrition: CRECER credit with education program in Bolivia Freedom from Hunger Research Paper 1999

[B35] Lagarde M Haines A Palmer N The impact of conditional cash transfers on health outcomes and use of health services in low and middle income countries Cochrane Database Syst Rev 2009 4 CD008137 19821444 10.1002/14651858.CD008137 PMC7177213

[B36] Lagarde M Palmer N The impact of user fees on access to health services in low- and middle-income countries Cochrane Database Syst Rev 2011 4 CD009094 21491414 10.1002/14651858.CD009094 PMC10025428

[B37] Lagarde M Palmer N The impact of user fees on health service utilization in low- and middle-income countries: how strong is the evidence? Bulletin of the World Health Organization 2008 86 11 839 848 10.2471/BLT.07.049197 19030689 PMC2649541

[B38] Walker N Fischer-Walker C Bryce J Bahl R Cousens S Standards for CHERG reviews of intervention effects on child survival Int J Epidemiol 2010 39 Suppl 1 i21 31 20348122 10.1093/ije/dyq036 PMC2845875

[B39] Gertler P Do conditional cash transfers improve child health? Evidence from PROGRESA's control randomized experiment The American Economic Review 2004 94 2 336 341 10.1257/0002828041302109 29068185

[B40] Abdu Z Mohammed Z Bashier I Eriksson B The impact of user fee exemption on service utilization and treatment seeking behaviour: the case of malaria in Sudan The International journal of health planning and management 2004 19 Suppl 1 S95 106 15686063 10.1002/hpm.777

[B41] Pitt MM Khandker SR Chowdhury OH Millimet DL Credit programs for the poor and the health status of children in rural bangladesh* International Economic Review 2003 44 1 87 118 10.1111/1468-2354.t01-1-00063

[B42] Lagarde M Barroy H Palmer N Assessing the effects of removing user fees in Zambia and Niger J Health Serv Res Policy 2012 17 1 30 36 10.1258/jhsrp.2011.010166 22096082

[B43] Attanasio O Gomez LC Heredia P Vera-Hernandez M The short-term impact of a conditional cash subsidy on child health and nutrition in Colombia Report Summary: Familias 2005 3

[B44] Barham T Maluccio JA Eradicating diseases: The effect of conditional cash transfers on vaccination coverage in rural Nicaragua Journal of Health Economics 2009 28 3 611 621 10.1016/j.jhealeco.2008.12.010 19233495

[B45] Gertler P Boyce S An Experiment in Incentive-based Welfare: The impact of Progresa on health in Mexico University of California, Berkeley 2001 30 37

[B46] Levin A Rahman MA Quayyum Z Routh S The demand for child curative care in two rural thanas of Bangladesh: effect of income and women&apos;s employment The International Journal of Health Planning and Management 2001 16 3 179 194 10.1002/hpm.630 11596556

[B47] Levy D Ohls J Evaluation of Jamaica’s PATH program: final report Report prepared 2007

[B48] Macours K Schady N Vakis R Cash Transfers, Behavioral Changes, and the Cognitive Development of Young Children: Evidence from a Randomized Experiment Policy Research Working Paper 2008 4759

[B49] Perova E Vakis R Welfare impacts of the “Juntos” Program in Peru: Evidence from a non-experimental evaluation The World Bank 2009

[B50] Robertson L Mushati P Eaton JW Dumba L Mavise G Makoni M Schumacher C Crea T Monasch R Sherr L Garnett GP Nyamukapa C Gregson S Effects of unconditional and conditional cash transfers on child health and development in Zimbabwe: a cluster-randomised trial Lancet 2013 381 1283 1292 23453283 10.1016/S0140-6736(12)62168-0 PMC3627205

[B51] Smith SC Village banking and maternal and child health: evidence from Ecuador and Honduras World Development 2002 30 4 707 723 10.1016/S0305-750X(01)00128-0

[B52] Burnham GM Pariyo G Galiwango E Wabwire-Mangen F Discontinuation of cost sharing in Uganda Bulletin of the World Health Organization 2004 82 3 187 195 15112007 PMC2585922

[B53] Dhillon RS Bonds MH Fraden M Ndahiro D Ruxin J The impact of reducing financial barriers on utilisation of a primary health care facility in Rwanda Glob Public Health 2012 7 1 71 86 10.1080/17441692.2011.593536 21732708 PMC3227794

