# Supplementary material for: Financial incentives and coverage of child health interventions: a systematic review and meta-analysis
Source: BMC Public Health. 2013 Sep 17;13(Suppl 3):S30. doi: 10.1186/1471-2458-13-S3-S30 (PMC3847540; doi:10.1186/1471-2458-13-S3-S30)
Supplement: Additional File 1 — Electronic search strategy for MEDLINE, EMBASE and AMED databases. [file 1471-2458-13-S3-S30-S1.pdf]

Additional File 1. Electronic search strategy for Medline, Embase and AMED databases

Cash transfer programs and voucher schemes

1. (cash adj3 transfer$).mp.
2. remove duplicates from 1
3. (voucher adj3 schem$).mp.
4. remove duplicates from 3
5. (demand adj2 side adj2 financ$).mp.
6. remove duplicates from 5
7. mchinji.mp.
8. remove duplicates from 7
9. dowa.mp.
10. remove duplicates from 9
11. zomba.mp.
12. remove duplicates from 11
13. (social adj2 grant$).mp.
14. remove duplicates from 13
15. (child$ adj3 support$ adj3 grant$).mp.
16. remove duplicates from 15
17. programa familia$.mp.
18. remove duplicates from 17
19. rural maintenance program$.mp.
20. rmp.mp.
21. bangladesh.mp.
22. 21 and 20
23. bolsa alimentacao.mp.
24. remove duplicates from 23
25. bolsa escola.mp.
26. remove duplicates from 25
27. bolsa familia.mp.
28. remove duplicates from 27
29. cartao alimentacao.mp.
30. remove duplicates from 29
31. (orphans and vulnerable children).mp. [mp=ti, ab, ot, nm, hw, ps, rs, ui, sh, tn, dm, mf, dv, kw]
32. burkina faso.mp.
33. 31 and 32
34. bono de desarrollo humano.mp.
35. remove duplicates from 34
36. progresa.mp.
37. remove duplicates from 36
38. janani suraksha yojana.mp.
39. remove duplicates from 38
40. jaring pengaman sosial.mp.
41. remove duplicates from 40
42. path.mp.
43. jamaica.mp.
44. 42 and 43
45. remove duplicates from 44
46. mdicp.mp.
47. remove duplicates from 46
48. (Malawi Diffusion and Ideational Change Project).mp. [mp=ti, ab, ot, nm, hw, ps, rs, ui, sh, tn, dm, mf, dv, kw]
49. remove duplicates from 48
50. 47 or 49

51. remove duplicates from 50
52. Programa de Apoyo Alimentario.mp.
53. remove duplicates from 52
54. PAL.mp.
55. mexico.mp.
56. 54 and 55
57. remove duplicates from 56
58. safe delivery incentive program$.mp.
59. remove duplicates from 58
60. sdip.mp.
61. 60 and nepal.mp.
62. remove duplicates from 61
63. 62 or 59
64. remove duplicates from 63
65. (cope and nigeria).mp.
66. (care of the poor and nigeria).mp.
67. 65 or 66
68. remove duplicates from 67
69. (child adj2 support adj2 program$).mp.
70. remove duplicates from 69
71. social risk mitigation project$.mp.
72. national voucher$ scheme.mp.
73. profamilia.mp.
74. remove duplicates from 73
75. remove duplicates from 72
76. local initiatives program$.mp.
77. remove duplicates from 76
78. (reproductive and child health program$).mp. [mp=ti, ab, ot, nm, hw, ps, rs, ui, sh, tn, dm, mf, dv, kw]
79. remove duplicates from 78
80. safe motherhood project$.mp.
81. remove duplicates from 80
82. maternal health voucher scheme.mp.
83. remove duplicates from 82
84. roll back malaria.mp.
85. child.mp. and 84
86. remove duplicates from 85
87. netmark.mp.
88. remove duplicates from 87
89. kinet.mp.
90. tanzania.mp.
91. 89 and 90
92. remove duplicates from 91
93. food stamp$.mp.
94. health.mp.
95. 93 and 94
96. world food program.mp.
97. remove duplicates from 95
98. remove duplicates from 96
99. (96 or 97 or 98) and 94
100. remove duplicates from 99
101. (social adj2 transfer$).mp.
102. remove duplicates from 101
103. (demand side adj2 finan$).mp.

104. remove duplicates from 103
105. (voucher adj3 program$).mp.
106. remove duplicates from 105
107. 31 and kenya.mp.
108. remove duplicates from 107
109. 2 or 4 or 6 or 8 or 10 or 12 or 14 or 16 or 18 or 19 or 22 or 24 or 26 or 28 or 30 or 33 or 35 or 37 or 39 or 41 or 45 or 51 or 53 or 57 or 64 or 68 or 70 or 71 or 74 or 75 or 77 or 79 or 81 or 83 or 86 or 88 or 92 or 97 or 98 or
110. remove duplicates from 109

Microcredit programs

1. (microcredit*.mp. or microfinanc*.mp. or (economic adj3 empowerment).mp. or microinsuranc*.mp. or micro-insuranc*.mp. or (micro adj3 insurance).mp.) and (evaluation*.mp. or impact*.mp.) and (health*.mp. or wellness.mp. or wellbeing.mp. or well-being.mp.)
2. remove duplicates from 112

User fee removal

Updated search using the search strategy conducted in: Lagarde M, Palmer N: The impact of user fees on access to health services in low- and middle-income countries. Cochrane database of systematic reviews (Online) 2011, 4:CD009094.
