# Supplementary material for: Financial incentives and coverage of child health interventions: a systematic review and meta-analysis
Source: BMC Public Health. 2013 Sep 17;13(Suppl 3):S30. doi: 10.1186/1471-2458-13-S3-S30 (PMC3847540; doi:10.1186/1471-2458-13-S3-S30)

1. Effect of financial incentives on percentage of newborns receiving colostrum

Study or Subgroup	Mean Difference	SE	Weight	Mean Difference IV, Random, 95% CI
**1.1.1 Conditional Microcredit**				
MkNelly 1998	0.28	0.0487	54.3%	0.28 [0.18, 0.38]
MkNelly 1999	0.14	0.0638	45.7%	0.14 [0.01, 0.27]
**Subtotal (95% CI)**			**100.0%**	**0.22 [0.08, 0.35]**

Heterogeneity: $Tau^2 = 0.01$; $Chi^2 = 3.04$, df = 1 (P = 0.08); $I^2 = 67\%$

Test for overall effect: Z = 3.10 (P = 0.002)

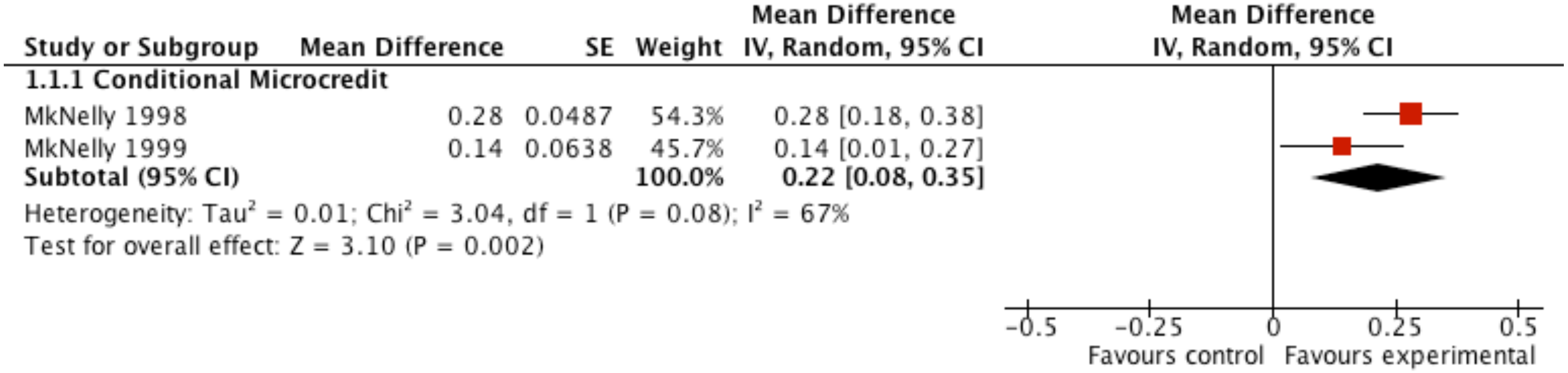

2. Effect of financial incentives on percentage of newborns breastfed within the first hour of life

Study or Subgroup	Mean Difference	SE	Weight	Mean Difference IV, Random, 95% CI	Mean Difference IV, Random, 95% CI
**1.2.1 Conditional Microcredit**					
MkNelly 1999	0.17	0.0807	100.0%	0.17 [0.01, 0.33]	
**Subtotal (95% CI)**			**100.0%**	**0.17 [0.01, 0.33]**	
Heterogeneity: Not applicable					
Test for overall effect: Z = 2.11 (P = 0.04)					

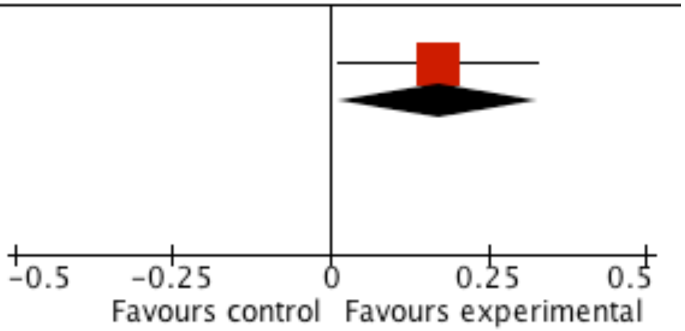

3. Effect of financial incentives on percentage of infants 0 to 5 months exclusively breastfed

Study or Subgroup	Mean Difference	SE	Weight	Mean Difference IV, Random, 95% CI
**1.3.1 Conditional Microcredit**				
MkNelly 1999	0.2	0.0844	100.0%	0.20 [0.03, 0.37]
**Subtotal (95% CI)**			**100.0%**	**0.20 [0.03, 0.37]**
Heterogeneity: Not applicable				
Test for overall effect: Z = 2.37 (P = 0.02)				

Mean Difference
IV, Random, 95% CI

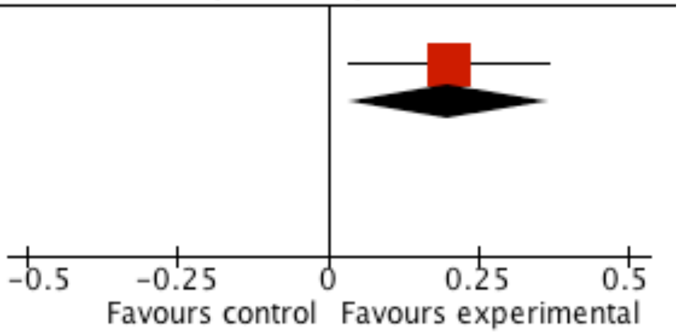

4. Effect of financial incentives on mean duration of exclusive breastfeeding

Study or Subgroup	Mean Difference	SE	Weight	Mean Difference IV, Random, 95% CI
**1.4.1 Conditional Microcredit**				
MkNelly 1998	11.487	5	100.0%	11.49 [1.69, 21.29]
**Subtotal (95% CI)**			**100.0%**	**11.49 [1.69, 21.29]**
Heterogeneity: Not applicable				
Test for overall effect: Z = 2.30 (P = 0.02)				

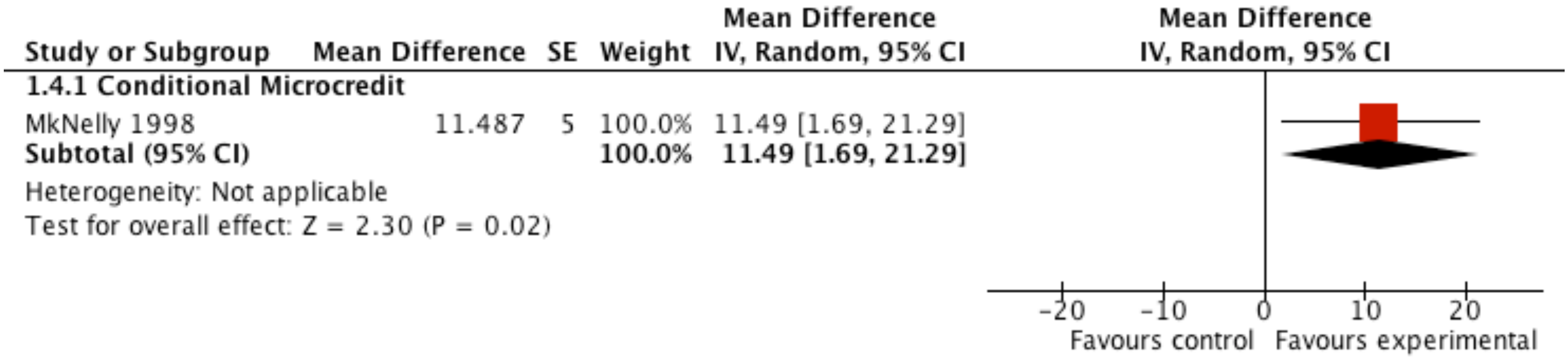

5. Effect of financial incentives on percentage of children <2 years of age currently or previously breastfed

Study or Subgroup	Mean Difference	SE	Weight	Mean Difference IV, Random, 95% CI
**1.5.1 Unconditional Microcredit**				
Smith 2002	−0.1179	0.0473	42.6%	−0.12 [−0.21, −0.03]
Smith 2002	−0.0185	0.0277	57.4%	−0.02 [−0.07, 0.04]
**Subtotal (95% CI)**			**100.0%**	**−0.06 [−0.16, 0.04]**
Heterogeneity: $Tau^2 = 0.00$; $Chi^2 = 3.29$, df = 1 (P = 0.07); $I^2 = 70\%$				
Test for overall effect: Z = 1.24 (P = 0.22)				
**1.5.2 Conditional Microcredit**				
Smith 2002	−0.0217	0.0122	46.7%	−0.02 [−0.05, 0.00]
Smith 2002	0.007	0.0097	53.3%	0.01 [−0.01, 0.03]
**Subtotal (95% CI)**			**100.0%**	**−0.01 [−0.03, 0.02]**
Heterogeneity: $Tau^2 = 0.00$; $Chi^2 = 3.39$, df = 1 (P = 0.07); $I^2 = 71\%$				
Test for overall effect: Z = 0.45 (P = 0.66)				

Mean Difference
IV, Random, 95% CI

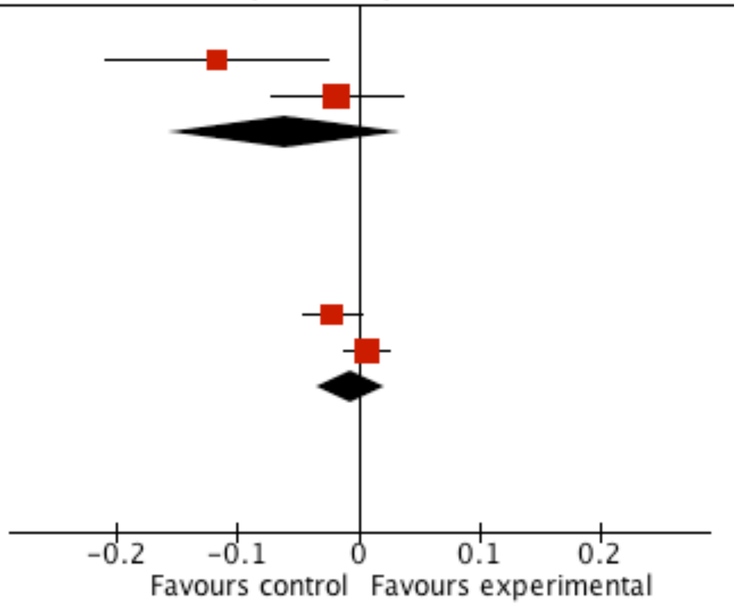

6. Effect of financial incentives on percentage of children receiving BCG vaccine

Study or Subgroup	Mean Difference	SE	Weight	Mean Difference IV, Random, 95% CI
**1.6.1 Conditional Cash Transfer**				
Barham 2009	0.0296	0.0202	35.7%	0.03 [−0.01, 0.07]
Levy 2007	−0.002	0.0046	56.0%	−0.00 [−0.01, 0.01]
Roy 2008	−0.11	0.0625	8.3%	−0.11 [−0.23, 0.01]
**Subtotal (95% CI)**			**100.0%**	**0.00 [−0.04, 0.04]**
Heterogeneity: $Tau^2 = 0.00$; $Chi^2 = 5.38$, df = 2 (P = 0.07); $I^2 = 63\%$				
Test for overall effect: Z = 0.01 (P = 0.99)				
**1.6.2 Conditional Microcredit**				
MkNelly 1998	0.09	0.0676	64.1%	0.09 [−0.04, 0.22]
MkNelly 1999	0.09	0.0904	35.9%	0.09 [−0.09, 0.27]
**Subtotal (95% CI)**			**100.0%**	**0.09 [−0.02, 0.20]**
Heterogeneity: $Tau^2 = 0.00$; $Chi^2 = 0.00$, df = 1 (P = 1.00); $I^2 = 0\%$				
Test for overall effect: Z = 1.66 (P = 0.10)				

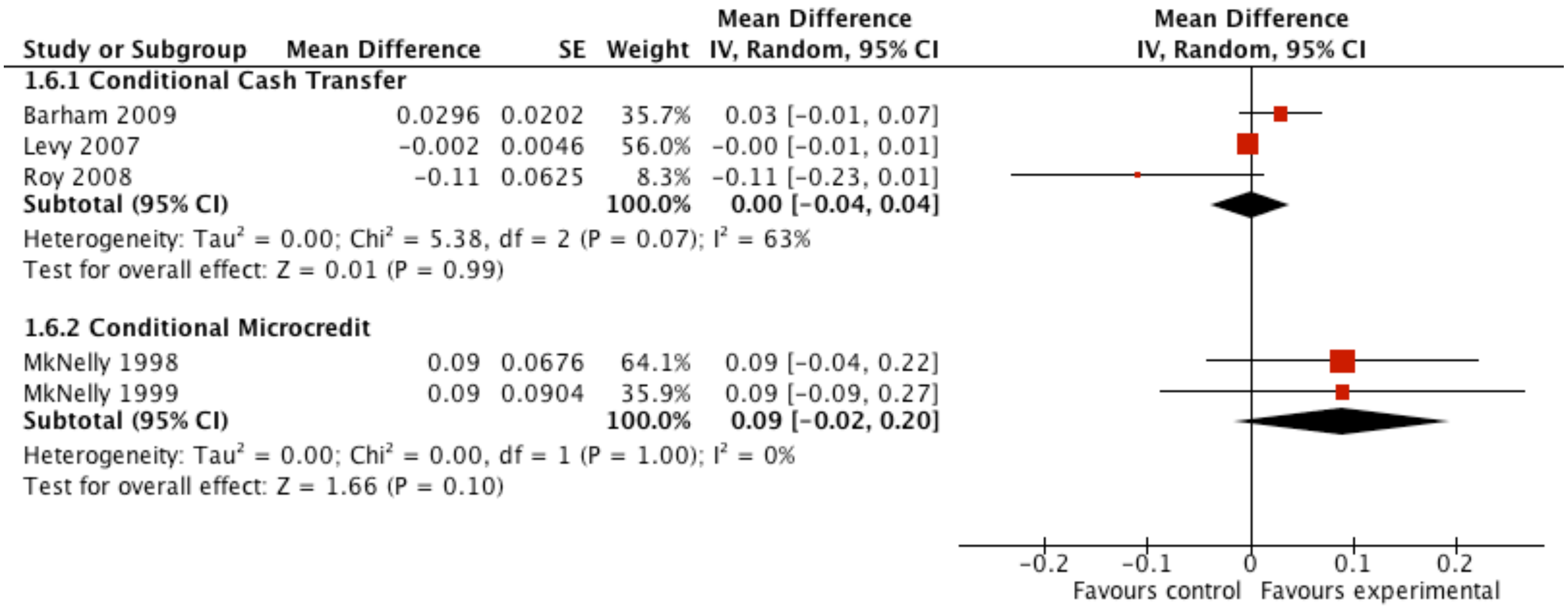

7. Effect of financial incentives on percentage of children receiving DPT-1 vaccine

Study or Subgroup	Mean Difference	SE	Weight	Mean Difference IV, Random, 95% CI
**1.7.1 Conditional Cash Transfer**				
Attanasio 2005	0.077	0.0342	74.2%	0.08 [0.01, 0.14]
Roy 2008	0.003	0.0618	25.8%	0.00 [−0.12, 0.12]
**Subtotal (95% CI)**			**100.0%**	**0.06 [−0.01, 0.12]**
Heterogeneity: $Tau^2 = 0.00$; $Chi^2 = 1.10$, $df = 1$ ($P = 0.29$); $I^2 = 9\%$				
Test for overall effect: $Z = 1.79$ ($P = 0.07$)				
**1.7.2 Unconditional Microcredit**				
Amin 2001	−0.019	0.0852	100.0%	−0.02 [−0.19, 0.15]
**Subtotal (95% CI)**			**100.0%**	**−0.02 [−0.19, 0.15]**
Heterogeneity: Not applicable				
Test for overall effect: $Z = 0.22$ ($P = 0.82$)				
**1.7.3 Conditional Microcredit**				
MkNelly 1998	−0.1148	0.069	54.1%	−0.11 [−0.25, 0.02]
MkNelly 1999	0.0991	0.0922	45.9%	0.10 [−0.08, 0.28]
**Subtotal (95% CI)**			**100.0%**	**−0.02 [−0.23, 0.19]**
Heterogeneity: $Tau^2 = 0.02$; $Chi^2 = 3.45$, $df = 1$ ($P = 0.06$); $I^2 = 71\%$				
Test for overall effect: $Z = 0.16$ ($P = 0.88$)				
**1.7.4 Conditional Voucher**				
Morris 2004	0.069	0.0301	100.0%	0.07 [0.01, 0.13]
**Subtotal (95% CI)**			**100.0%**	**0.07 [0.01, 0.13]**
Heterogeneity: Not applicable				
Test for overall effect: $Z = 2.29$ ($P = 0.02$)				

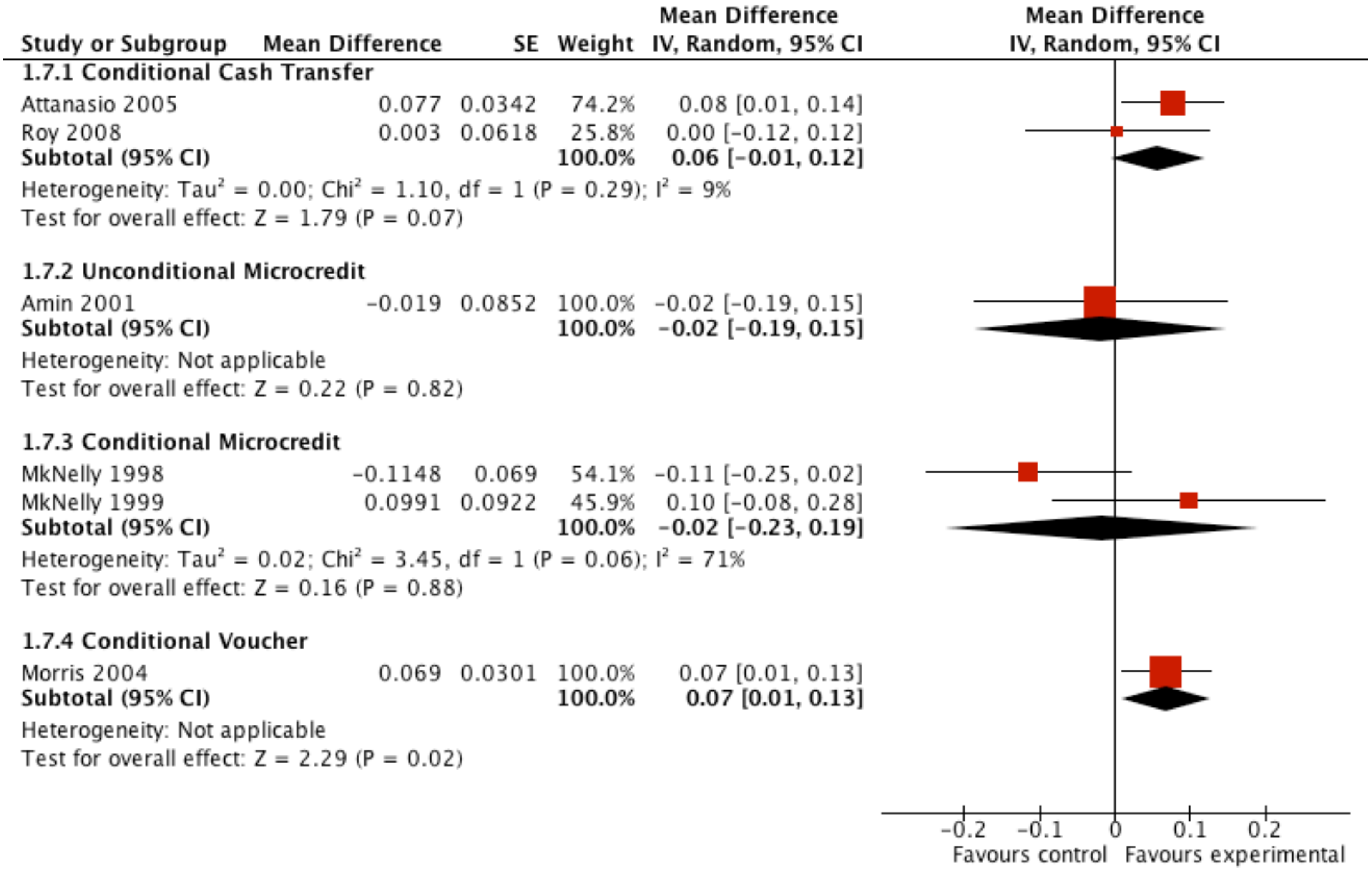

8. Effect of financial incentives on percentage of children receiving DPT-3 vaccine

Study or Subgroup	Mean Difference	SE	Weight	Mean Difference IV, Random, 95% CI
**1.8.1 Conditional Cash Transfer**				
Barham 2009	0.1	0.0612	13.0%	0.10 [−0.02, 0.22]
Levy 2007	0.0059	0.0048	75.4%	0.01 [−0.00, 0.02]
Roy 2008	−0.035	0.0657	11.6%	−0.04 [−0.16, 0.09]
**Subtotal (95% CI)**			**100.0%**	**0.01 [−0.03, 0.06]**
Heterogeneity: $Tau^2 = 0.00$; $Chi^2 = 2.75$, df = 2 (P = 0.25); $I^2 = 27\%$				
Test for overall effect: Z = 0.55 (P = 0.58)				
**1.8.2 Conditional Microcredit**				
MkNelly 1998	−0.08	0.069	53.6%	−0.08 [−0.22, 0.06]
MkNelly 1999	0.16	0.0943	46.4%	0.16 [−0.02, 0.34]
**Subtotal (95% CI)**			**100.0%**	**0.03 [−0.20, 0.27]**
Heterogeneity: $Tau^2 = 0.02$; $Chi^2 = 4.22$, df = 1 (P = 0.04); $I^2 = 76\%$				
Test for overall effect: Z = 0.26 (P = 0.79)				

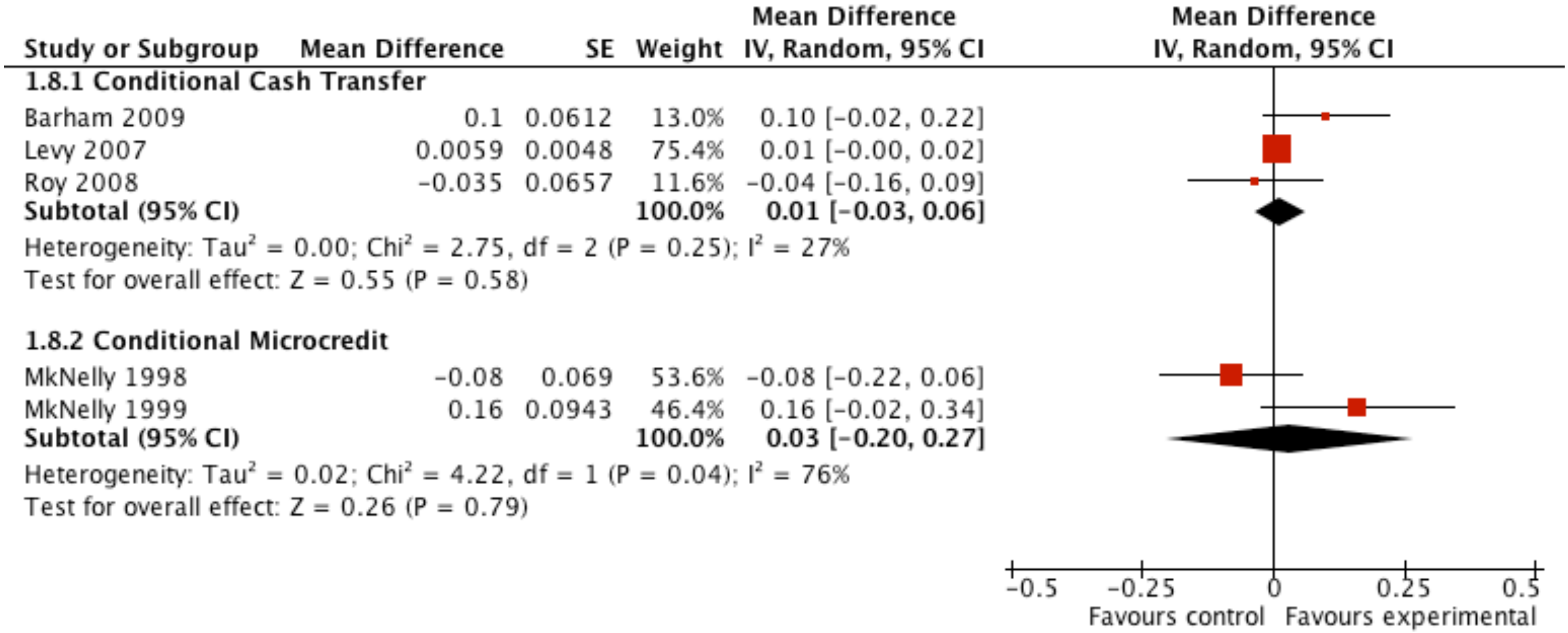

9. Effect of financial incentives on percentage of children receiving measles vaccine (MCV)

Study or Subgroup	Mean Difference	SE	Weight	Mean Difference IV, Random, 95% CI
**1.9.1 Conditional Cash Transfer**				
Barham 2009	0.09	0.051	30.6%	0.09 [−0.01, 0.19]
Levy 2007	0.013	0.012	43.2%	0.01 [−0.01, 0.04]
Roy 2008	−0.1506	0.0633	26.2%	−0.15 [−0.27, −0.03]
**Subtotal (95% CI)**			**100.0%**	**−0.01 [−0.11, 0.09]**
Heterogeneity: $Tau^2 = 0.01$; $Chi^2 = 8.94$, df = 2 (P = 0.01); $I^2 = 78\%$				
Test for overall effect: Z = 0.13 (P = 0.90)				
**1.9.2 Unconditional Microcredit**				
Amin 2001	0.094	0.0065	100.0%	0.09 [0.08, 0.11]
**Subtotal (95% CI)**			**100.0%**	**0.09 [0.08, 0.11]**
Heterogeneity: Not applicable				
Test for overall effect: Z = 14.46 (P < 0.00001)				
**1.9.3 Conditional Microcredit**				
MkNelly 1998	−0.25	0.0754	50.4%	−0.25 [−0.40, −0.10]
MkNelly 1999	0.18	0.084	49.6%	0.18 [0.02, 0.34]
**Subtotal (95% CI)**			**100.0%**	**−0.04 [−0.46, 0.38]**
Heterogeneity: $Tau^2 = 0.09$; $Chi^2 = 14.51$, df = 1 (P = 0.0001); $I^2 = 93\%$				
Test for overall effect: Z = 0.17 (P = 0.86)				
**1.9.4 Conditional Voucher**				
Morris 2004	−0.002	0.0469	100.0%	−0.00 [−0.09, 0.09]
**Subtotal (95% CI)**			**100.0%**	**−0.00 [−0.09, 0.09]**
Heterogeneity: Not applicable				
Test for overall effect: Z = 0.04 (P = 0.97)				

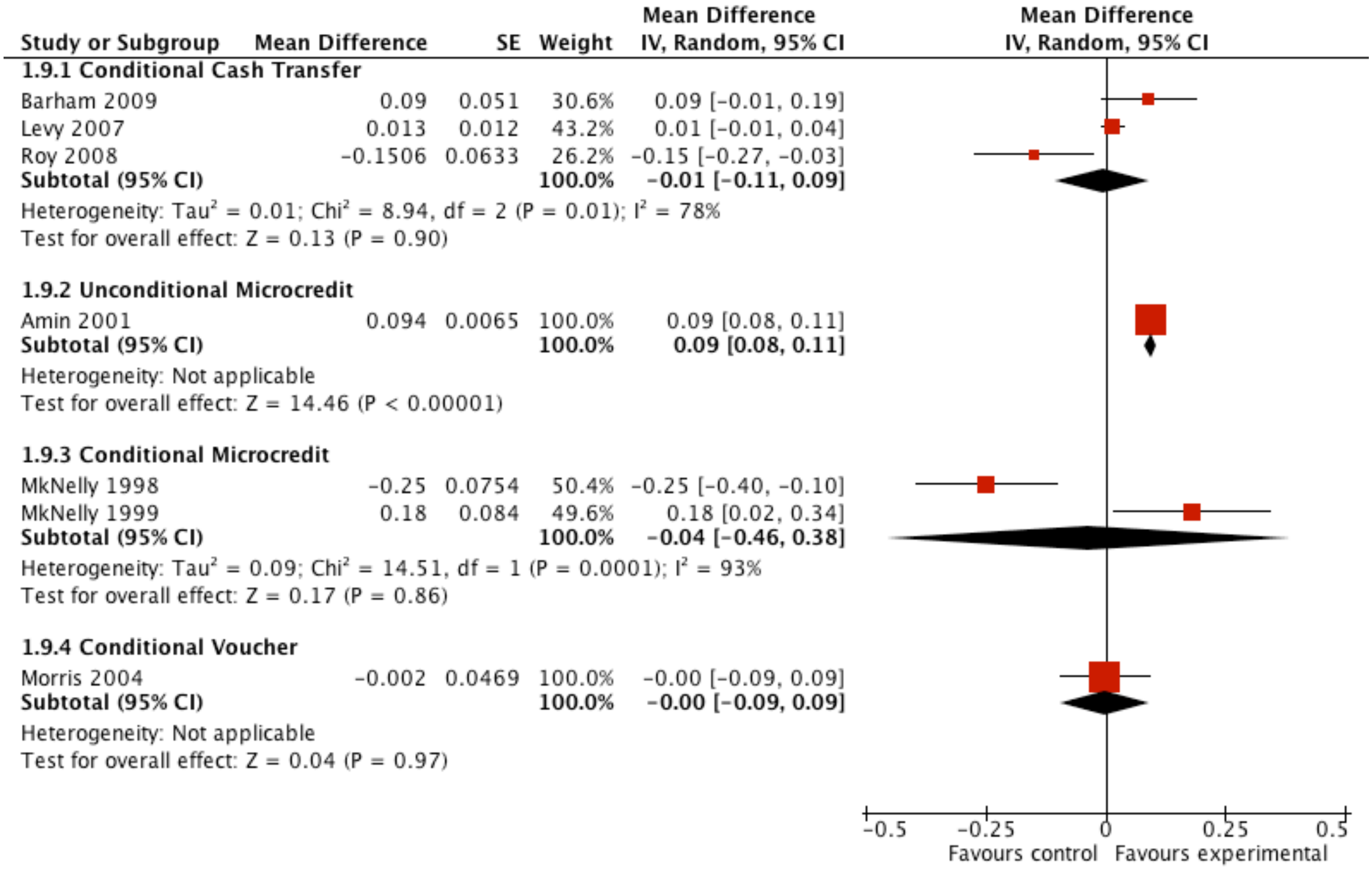

10. Effect of financial incentives on percentage of children receiving OPV-3 vaccine

Study or Subgroup	Mean Difference	SE	Weight	Mean Difference IV, Random, 95% CI
**1.10.1 Conditional Cash Transfer**				
Barham 2009	0.1	0.0459	38.9%	0.10 [0.01, 0.19]
Levy 2007	0.0129	0.05	35.4%	0.01 [−0.09, 0.11]
Roy 2008	−0.034	0.0642	25.8%	−0.03 [−0.16, 0.09]
**Subtotal (95% CI)**			**100.0%**	**0.03 [−0.04, 0.11]**
Heterogeneity: $Tau^2 = 0.00$; $Chi^2 = 3.33$, df = 2 (P = 0.19); $I^2 = 40\%$				
Test for overall effect: Z = 0.88 (P = 0.38)				
**1.10.2 Conditional Microcredit**				
MkNelly 1998	−0.05	0.084	41.7%	−0.05 [−0.21, 0.11]
MkNelly 1999	−0.09	0.071	58.3%	−0.09 [−0.23, 0.05]
**Subtotal (95% CI)**			**100.0%**	**−0.07 [−0.18, 0.03]**
Heterogeneity: $Tau^2 = 0.00$; $Chi^2 = 0.13$, df = 1 (P = 0.72); $I^2 = 0\%$				
Test for overall effect: Z = 1.35 (P = 0.18)				

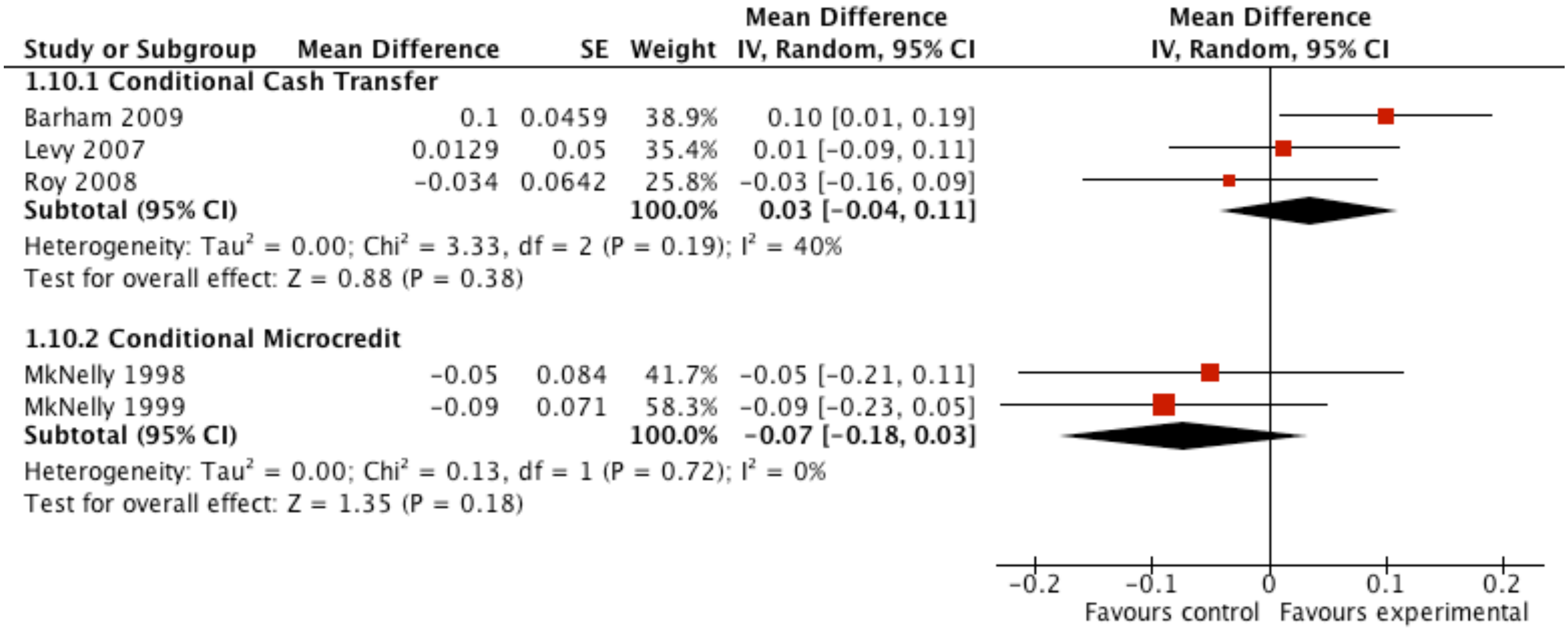

11. Effect of financial incentives on percentage of children receiving any vaccine

Study or Subgroup	Mean Difference	SE	Weight	Mean Difference IV, Random, 95% CI
**1.11.1 Conditional Cash Transfer**				
Perova 2009	0.22	0.05	100.0%	0.22 [0.12, 0.32]
**Subtotal (95% CI)**			**100.0%**	**0.22 [0.12, 0.32]**
Heterogeneity: Not applicable				
Test for overall effect: Z = 4.40 (P < 0.0001)				
**1.11.2 Unconditional Microcredit**				
Montgomery 2011	0.0816	0.0441	100.0%	0.08 [−0.00, 0.17]
**Subtotal (95% CI)**			**100.0%**	**0.08 [−0.00, 0.17]**
Heterogeneity: Not applicable				
Test for overall effect: Z = 1.85 (P = 0.06)				
**1.11.3 Conditional Microcredit**				
MkNelly 1998	−0.07	0.0655	52.5%	−0.07 [−0.20, 0.06]
MkNelly 1999	0.21	0.0909	47.5%	0.21 [0.03, 0.39]
**Subtotal (95% CI)**			**100.0%**	**0.06 [−0.21, 0.34]**
Heterogeneity: $Tau^2 = 0.03$; $Chi^2 = 6.25$, df = 1 (P = 0.01); $I^2 = 84\%$				
Test for overall effect: Z = 0.45 (P = 0.65)				

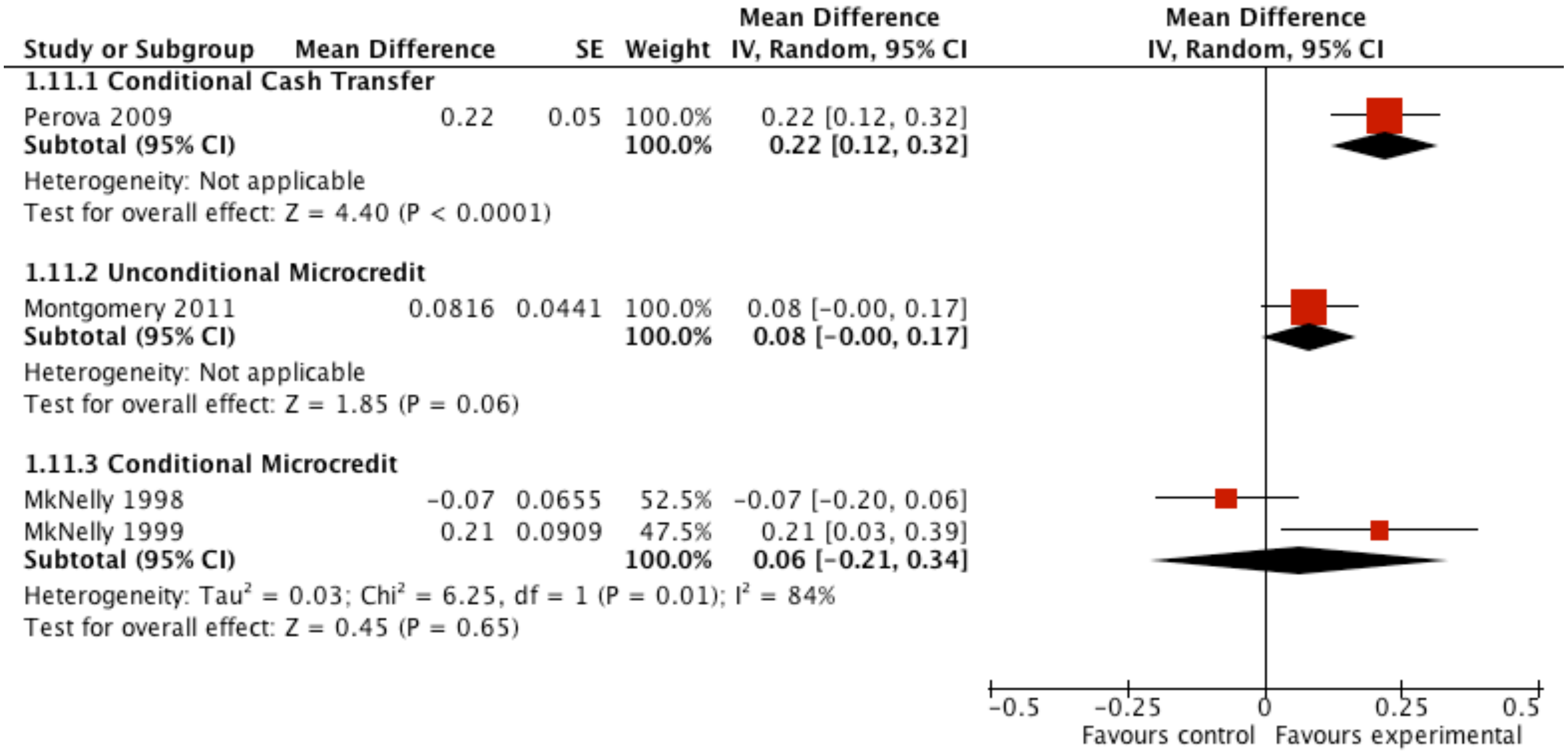

12. Effect of financial incentives on percentage of children receiving their full EPI vaccine schedule

Study or Subgroup	Mean Difference	SE	Weight	Mean Difference IV, Random, 95% CI
**1.12.1 Unconditional Cash Transfer**				
Robertson 2012	0.031	0.0352	100.0%	0.03 [−0.04, 0.10]
**Subtotal (95% CI)**			**100.0%**	**0.03 [−0.04, 0.10]**
Heterogeneity: Not applicable				
Test for overall effect: Z = 0.88 (P = 0.38)				
**1.12.2 Conditional Cash Transfer**				
Barham 2009	0.14	0.0612	19.8%	0.14 [0.02, 0.26]
Maluccio 2004	0.061	0.102	7.4%	0.06 [−0.14, 0.26]
Robertson 2012	0.018	0.0347	55.4%	0.02 [−0.05, 0.09]
Roy 2008	0.021	0.0653	17.5%	0.02 [−0.11, 0.15]
**Subtotal (95% CI)**			**100.0%**	**0.05 [−0.01, 0.10]**
Heterogeneity: $Tau^2 = 0.00$; $Chi^2 = 3.17$, df = 3 (P = 0.37); $I^2 = 5\%$				
Test for overall effect: Z = 1.64 (P = 0.10)				

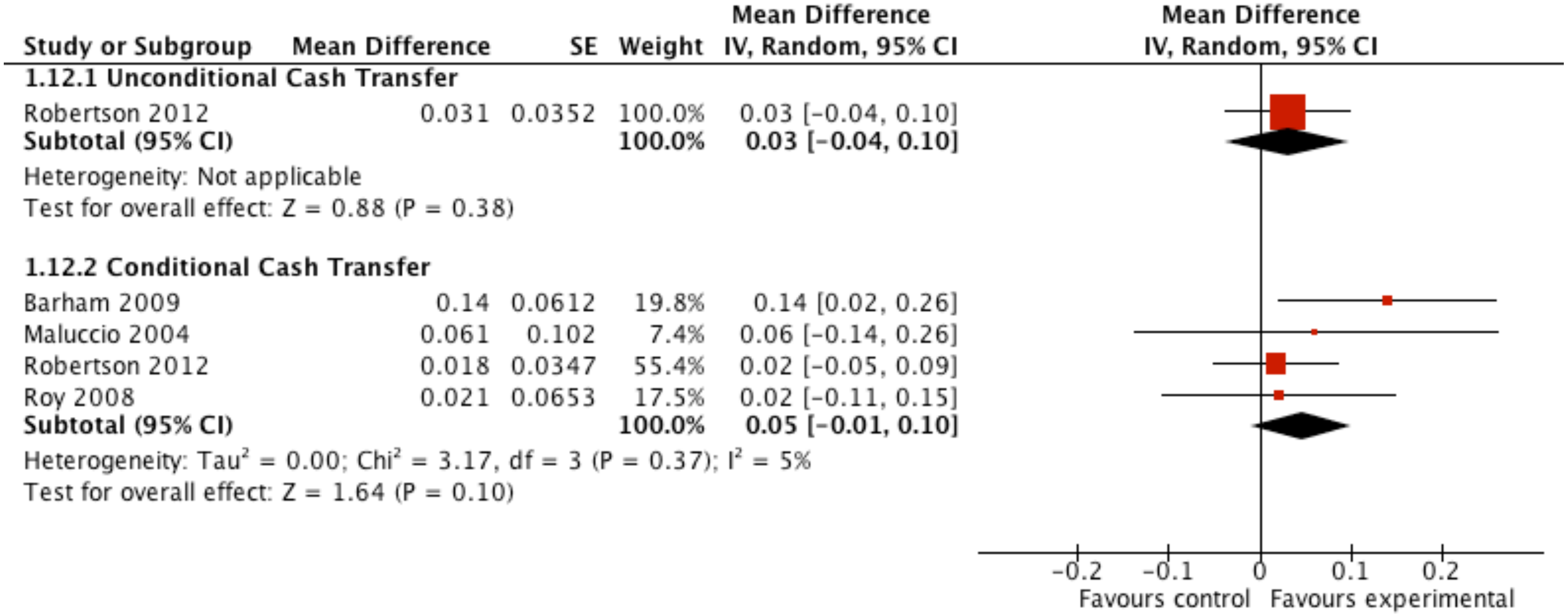

13. Effect of financial incentives on percentage of children accessing preventive health care in the previous 6 months

Study or Subgroup	Mean Difference	SE	Weight	Mean Difference IV, Random, 95% CI
**1.13.1 Unconditional Cash Transfer**				
Paxson 2010	0.01	0.0561	100.0%	0.01 [−0.10, 0.12]
**Subtotal (95% CI)**			**100.0%**	**0.01 [−0.10, 0.12]**
Heterogeneity: Not applicable				
Test for overall effect: Z = 0.18 (P = 0.86)				
**1.13.2 Conditional Cash Transfer**				
Attanasio 2005	0.25	0.0561	18.9%	0.25 [0.14, 0.36]
Galasso 2001	0	0.0153	21.0%	0.00 [−0.03, 0.03]
Macours 2008	−0.004	0.02	20.9%	−0.00 [−0.04, 0.04]
Maluccio 2004	0.11	0.059	18.7%	0.11 [−0.01, 0.23]
Perova 2009	0.37	0.03	20.5%	0.37 [0.31, 0.43]
**Subtotal (95% CI)**			**100.0%**	**0.14 [−0.00, 0.29]**
Heterogeneity: $Tau^2 = 0.03$; $Chi^2 = 144.58$, df = 4 (P < 0.00001); $I^2 = 97\%$				
Test for overall effect: Z = 1.93 (P = 0.05)				
**1.13.3 Unconditional Microcredit**				
Amin 2001	0.0421	0.0101	100.0%	0.04 [0.02, 0.06]
**Subtotal (95% CI)**			**100.0%**	**0.04 [0.02, 0.06]**
Heterogeneity: Not applicable				
Test for overall effect: Z = 4.17 (P < 0.0001)				
**1.13.4 Conditional Voucher**				
Morris 2004	0.156	0.014	100.0%	0.16 [0.13, 0.18]
**Subtotal (95% CI)**			**100.0%**	**0.16 [0.13, 0.18]**
Heterogeneity: Not applicable				
Test for overall effect: Z = 11.14 (P < 0.00001)				

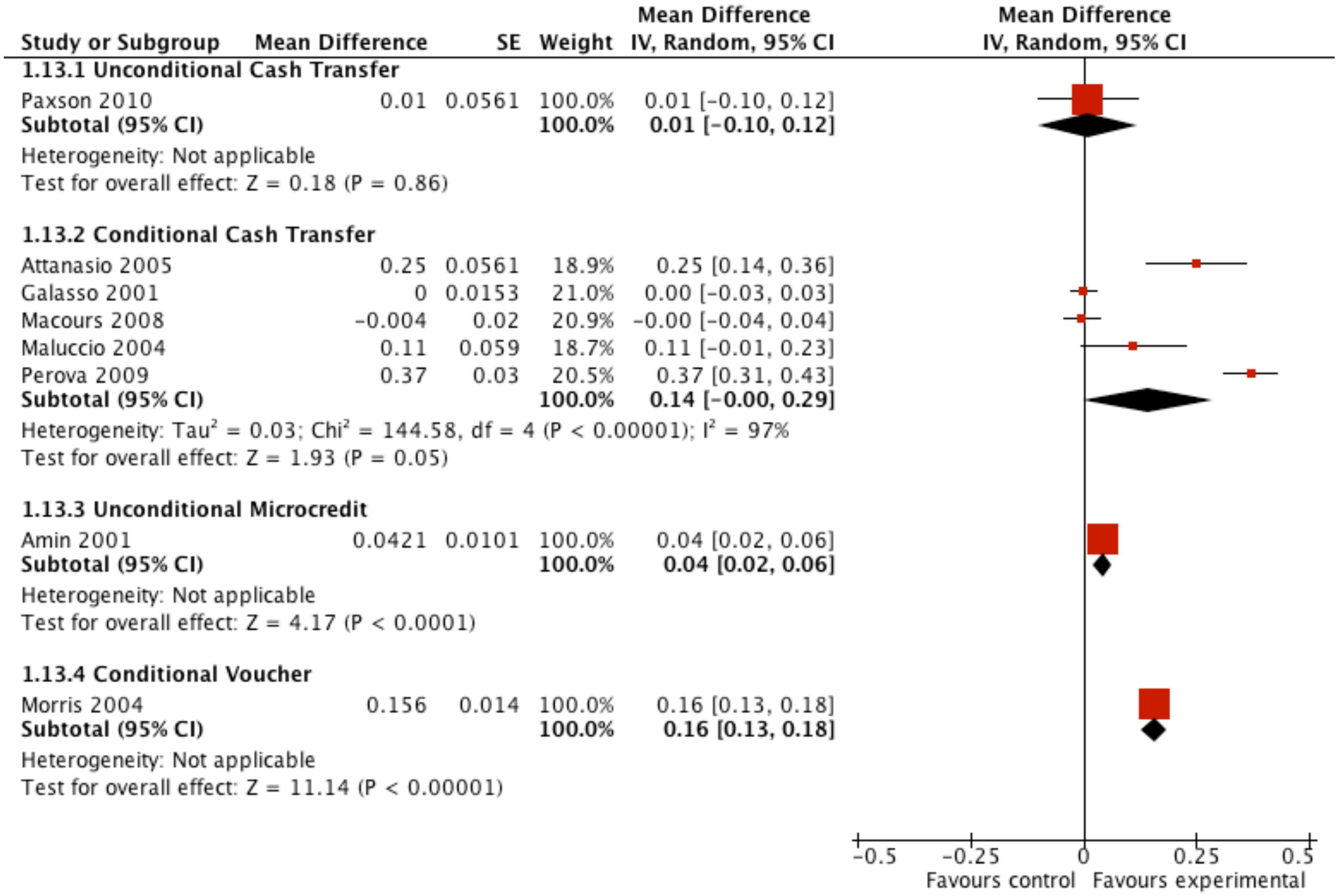

14. Effect of financial incentives on percentage of children accessing curative health care in the previous 6 months

Study or Subgroup	Mean Difference	SE	Weight	Mean Difference IV, Random, 95% CI
**1.14.1 Conditional Cash Transfer**				
Perova 2009	0.22	0.05	100.0%	0.22 [0.12, 0.32]
**Subtotal (95% CI)**			**100.0%**	**0.22 [0.12, 0.32]**
Heterogeneity: Not applicable				
Test for overall effect: Z = 4.40 (P < 0.0001)				
**1.14.2 Unconditional Microcredit**				
Levin 2001	0.0679	0.0375	18.6%	0.07 [−0.01, 0.14]
Montgomery 2011	0.1041	0.0179	81.4%	0.10 [0.07, 0.14]
**Subtotal (95% CI)**			**100.0%**	**0.10 [0.07, 0.13]**
Heterogeneity: $Tau^2 = 0.00$; $Chi^2 = 0.76$, df = 1 (P = 0.38); $I^2 = 0\%$				
Test for overall effect: Z = 6.03 (P < 0.00001)				
**1.14.3 User Fee Removal**				
Abdu 2004	0.28	0.0372	44.9%	0.28 [0.21, 0.35]
Dhillon 2012	0.3762	0.0211	55.1%	0.38 [0.33, 0.42]
**Subtotal (95% CI)**			**100.0%**	**0.33 [0.24, 0.43]**
Heterogeneity: $Tau^2 = 0.00$; $Chi^2 = 5.06$, df = 1 (P = 0.02); $I^2 = 80\%$				
Test for overall effect: Z = 6.96 (P < 0.00001)				

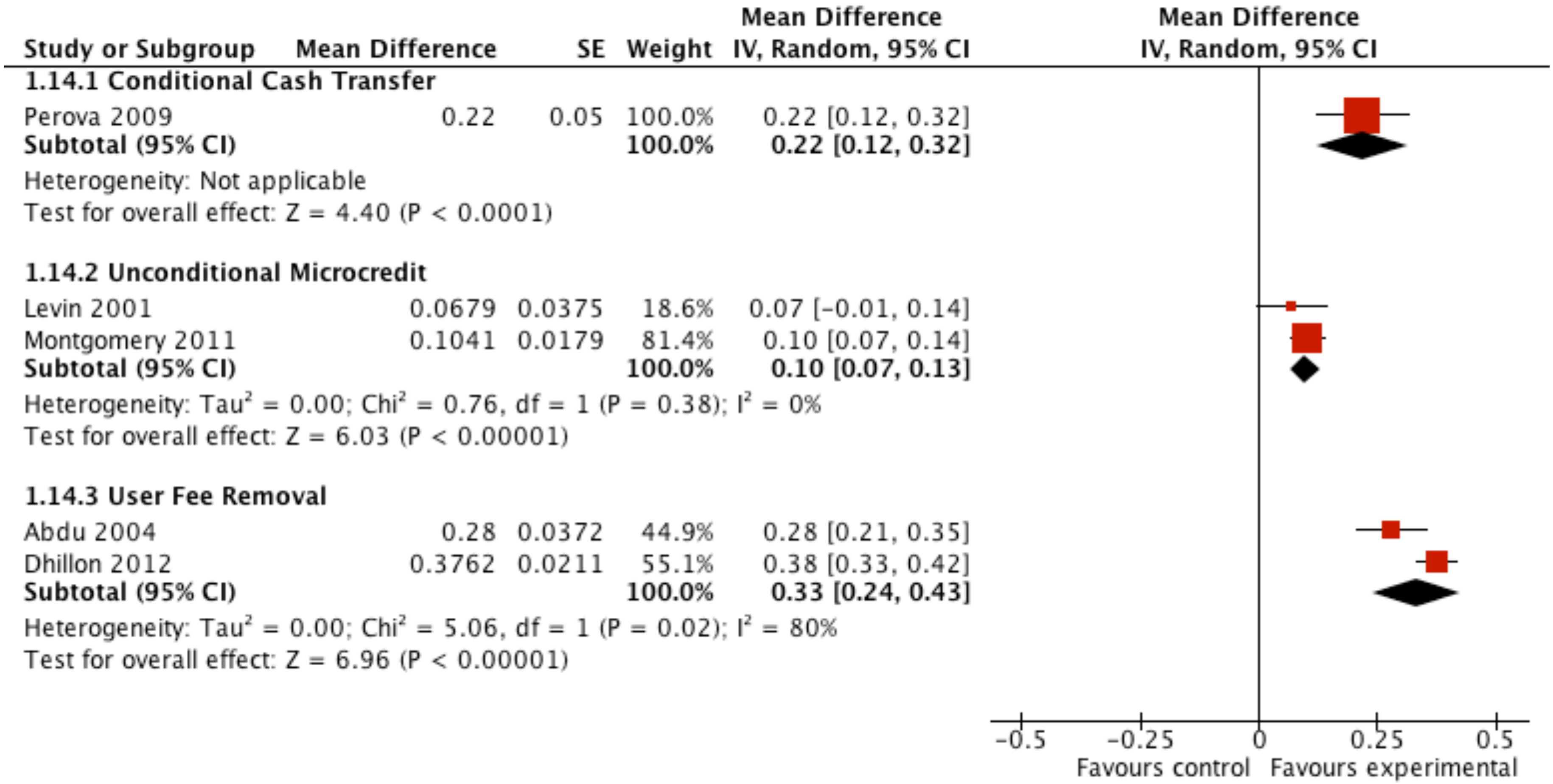

15. Effect of financial incentives on percentage of children accessing any health care in the previous 6 months

Study or Subgroup	Mean Difference	SE	Weight	Mean Difference IV, Random, 95% CI
**1.15.1 Conditional Cash Transfer**				
Brazil 2005	0.0426	0.0303	100.0%	0.04 [-0.02, 0.10]
**Subtotal (95% CI)**			**100.0%**	**0.04 [-0.02, 0.10]**
Heterogeneity: Not applicable				
Test for overall effect: Z = 1.41 (P = 0.16)				

Mean Difference IV, Random, 95% CI

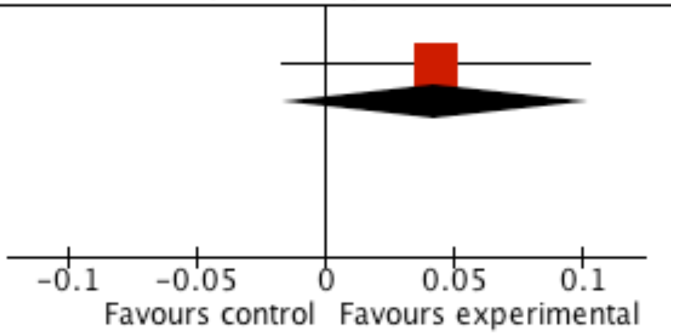

16. Effect of financial incentives on mean number of preventive child health care visits in the previous month

Study or Subgroup	Mean Difference	SE	Weight	Mean Difference IV, Random, 95% CI	Mean Difference IV, Random, 95% CI
**1.16.1 Conditional Cash Transfer**					
Levy 2007	0.3835	0.119	100.0%	0.38 [0.15, 0.62]	
**Subtotal (95% CI)**			**100.0%**	**0.38 [0.15, 0.62]**	
Heterogeneity: Not applicable					
Test for overall effect: Z = 3.22 (P = 0.001)					
**1.16.2 User Fee Removal**					
Wilkinson 2001	-0.028	0.08	100.0%	-0.03 [-0.18, 0.13]	
**Subtotal (95% CI)**			**100.0%**	**-0.03 [-0.18, 0.13]**	
Heterogeneity: Not applicable					
Test for overall effect: Z = 0.35 (P = 0.73)					

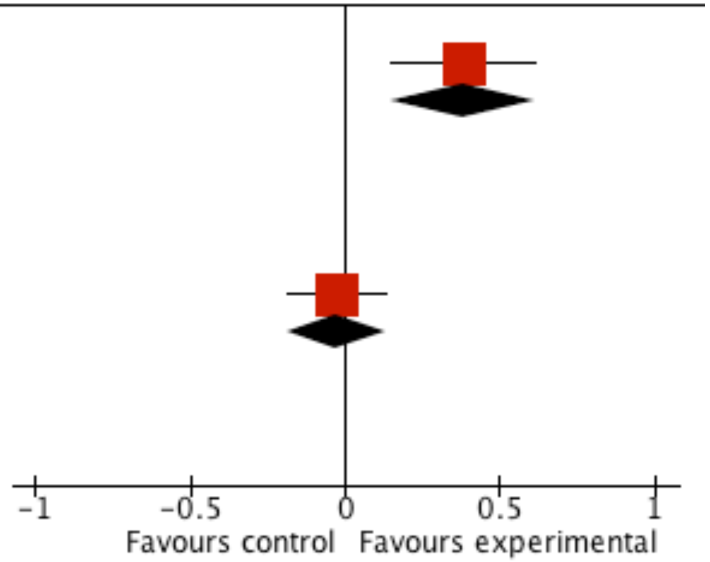

17. Effect of financial incentives on mean number of curative child health care visits in the previous month

Study or Subgroup	Mean Difference	SE	Weight	Mean Difference IV, Random, 95% CI
**1.17.1 User Fee Removal**				
Burgert 2011	1.19	0.1888	34.7%	1.19 [0.82, 1.56]
Lagarde 2012	0.89	0.09	65.3%	0.89 [0.71, 1.07]
**Subtotal (95% CI)**			**100.0%**	**0.99 [0.71, 1.27]**

Heterogeneity: $Tau^2 = 0.02$; $Chi^2 = 2.06$, df = 1 (P = 0.15); $I^2 = 51\%$

Test for overall effect: Z = 6.96 (P < 0.00001)

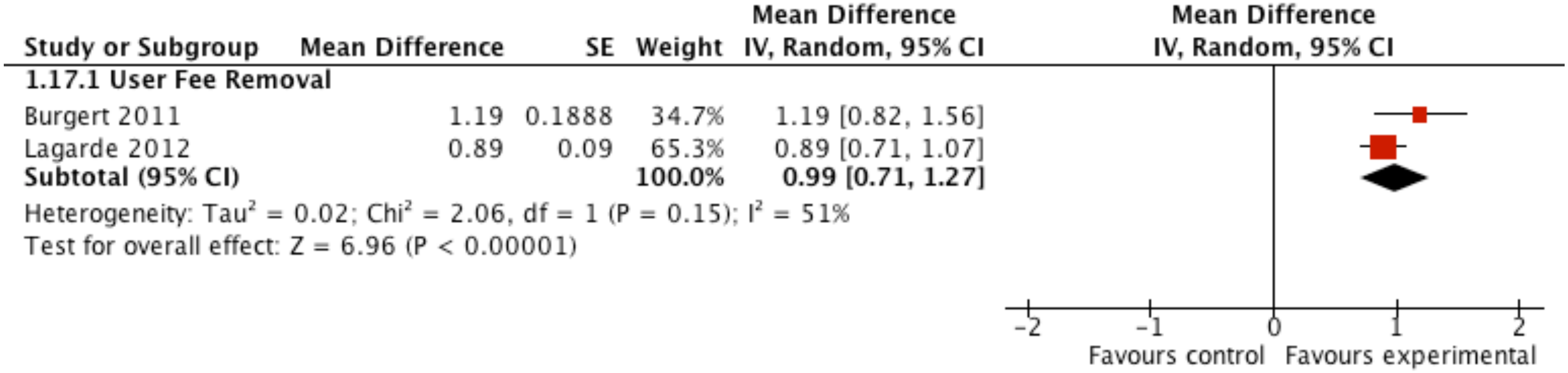

18. Effect of financial incentives on mean number of new child health care visits in the previous month

Study or Subgroup	Mean Difference	SE	Weight	Mean Difference IV, Random, 95% CI	Mean Difference IV, Random, 95% CI
**1.18.1 User Fee Removal**					
Burnham 2004	0.273	0.05	100.0%	0.27 [0.18, 0.37]	
**Subtotal (95% CI)**			**100.0%**	**0.27 [0.18, 0.37]**	
Heterogeneity: Not applicable					
Test for overall effect: Z = 5.46 (P < 0.00001)					

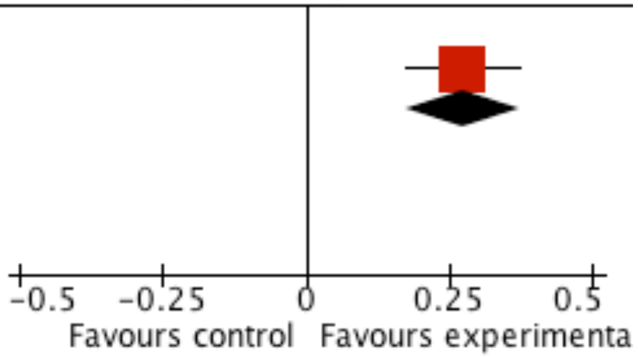

19. Effect of financial incentives on mean number of follow-up child health care visits in the previous month

Study or Subgroup	Mean Difference	SE	Weight	Mean Difference IV, Random, 95% CI	Mean Difference IV, Random, 95% CI
**1.19.1 User Fee Removal**					
Burnham 2004	0.813	0.0446	100.0%	0.81 [0.73, 0.90]	
**Subtotal (95% CI)**			**100.0%**	**0.81 [0.73, 0.90]**	
Heterogeneity: Not applicable					
Test for overall effect: Z = 18.23 (P < 0.00001)					

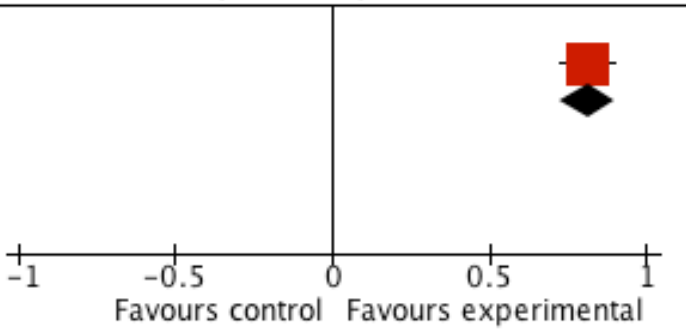

20. Effect of financial incentives on mean number of any child health care visits in the previous month

Study or Subgroup	Mean Difference	SE	Weight	Mean Difference IV, Random, 95% CI
**1.20.1 Conditional Cash Transfer**				
Gertler 2001	-0.01	0.0051	100.0%	-0.01 [-0.02, -0.00]
**Subtotal (95% CI)**			**100.0%**	**-0.01 [-0.02, -0.00]**
Heterogeneity: Not applicable				
Test for overall effect: Z = 1.96 (P = 0.05)				
**1.20.2 User Fee Removal**				
Burnham 2004	0.195	0.0471	100.0%	0.20 [0.10, 0.29]
**Subtotal (95% CI)**			**100.0%**	**0.20 [0.10, 0.29]**
Heterogeneity: Not applicable				
Test for overall effect: Z = 4.14 (P < 0.0001)				

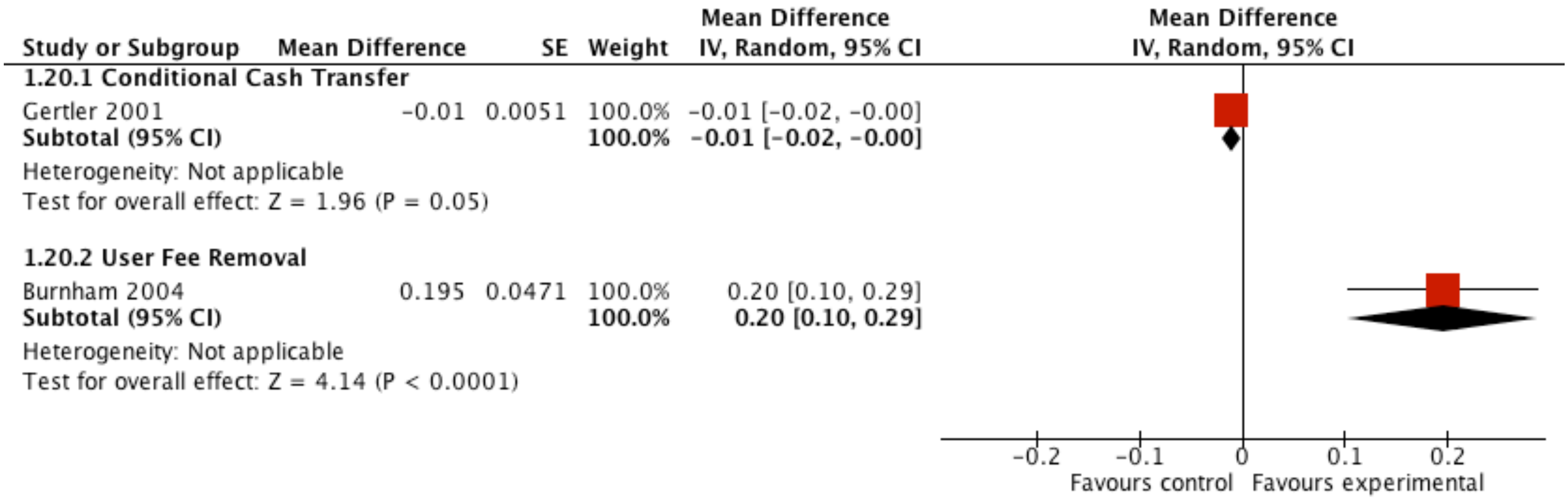

21. Effect of financial incentives on percentage of children receiving oral rehydration solution during their last diarrhoea episode

Study or Subgroup	Mean Difference	SE	Weight	Mean Difference IV, Random, 95% CI
**1.21.1 Unconditional Microcredit**				
Montgomery 2011	0.0179	0.0176	100.0%	0.02 [−0.02, 0.05]
**Subtotal (95% CI)**			**100.0%**	**0.02 [−0.02, 0.05]**
Heterogeneity: Not applicable				
Test for overall effect: Z = 1.02 (P = 0.31)				
**1.21.2 Conditional Microcredit**				
MkNelly 1998	0.65	0.06	100.0%	0.65 [0.53, 0.77]
**Subtotal (95% CI)**			**100.0%**	**0.65 [0.53, 0.77]**
Heterogeneity: Not applicable				
Test for overall effect: Z = 10.83 (P < 0.00001)				

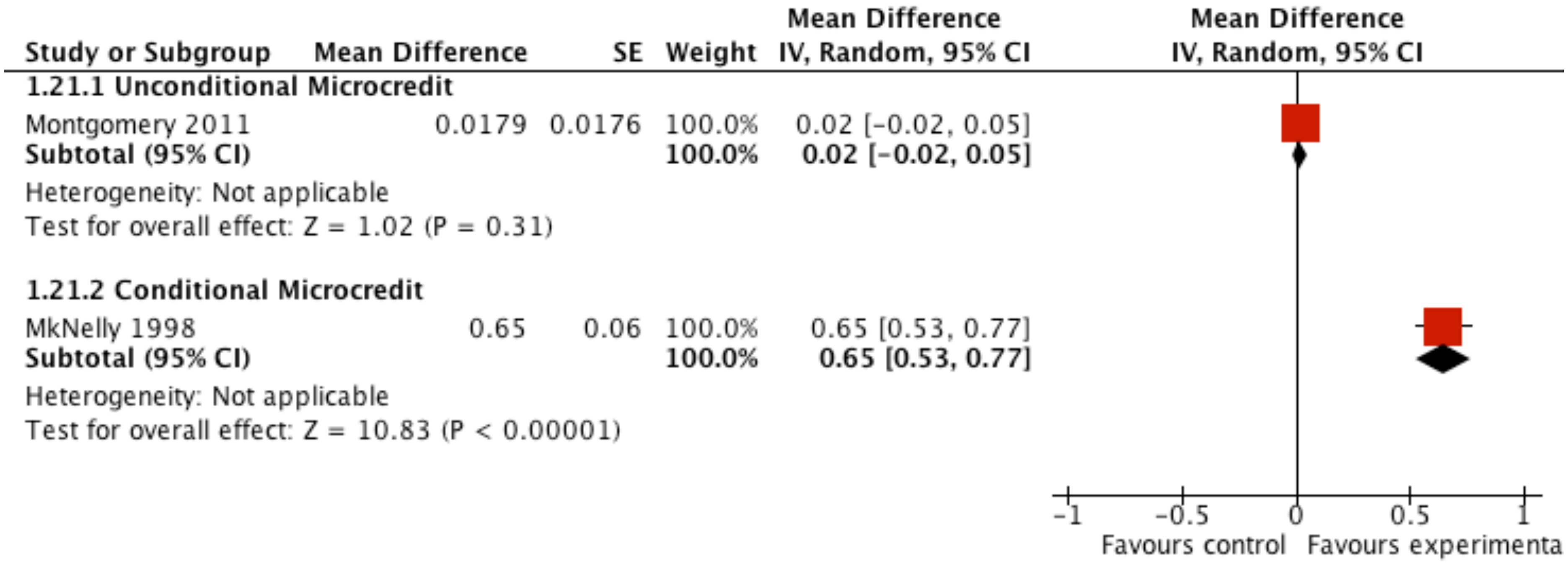

22. Effect of financial incentives on percentage of children continuing feeding during their last diarrhoea episode

Study or Subgroup	Mean Difference	SE	Weight	Mean Difference IV, Random, 95% CI
**1.22.1 Conditional Microcredit**				
MkNelly 1998	0.02	0.0638	61.0%	0.02 [−0.11, 0.15]
MkNelly 1999	0.04	0.0798	39.0%	0.04 [−0.12, 0.20]
**Subtotal (95% CI)**			**100.0%**	**0.03 [−0.07, 0.13]**

Heterogeneity: $Tau^2 = 0.00$; $Chi^2 = 0.04$, df = 1 (P = 0.84); $I^2 = 0\%$
Test for overall effect: Z = 0.56 (P = 0.58)

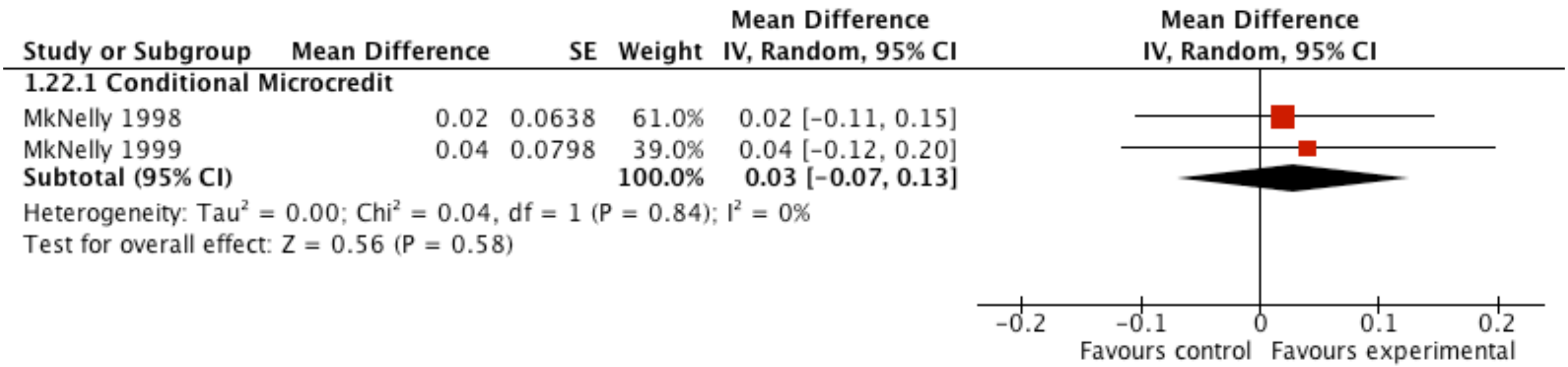

23. Effect of financial incentives on percentage of children taken to a health facility during their last episode of diarrhoea

Study or Subgroup	Mean Difference	SE	Weight	Mean Difference IV, Random, 95% CI	Mean Difference IV, Random, 95% CI
**1.23.1 Conditional Cash Transfer**					
Macours 2008	0.027	0.03	100.0%	0.03 [-0.03, 0.09]	
**Subtotal (95% CI)**			**100.0%**	**0.03 [-0.03, 0.09]**	
Heterogeneity: Not applicable					
Test for overall effect: Z = 0.90 (P = 0.37)					

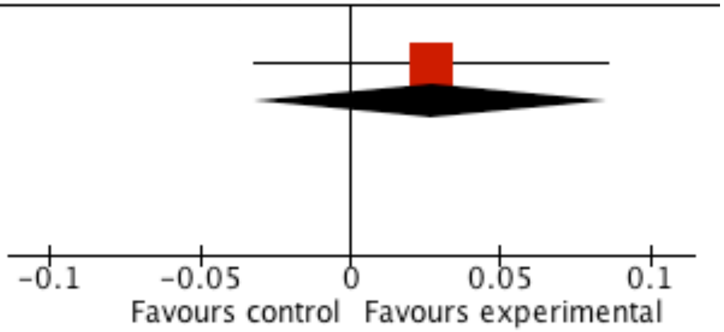

24. Effect of financial incentives on percentage of children receiving deworming drugs in the previous 6 months

Study or Subgroup	Mean Difference	SE	Weight	Mean Difference IV, Random, 95% CI
**1.24.1 Unconditional Cash Transfer**				
Paxson 2010	0.08	0.0357	100.0%	0.08 [0.01, 0.15]
**Subtotal (95% CI)**			**100.0%**	**0.08 [0.01, 0.15]**
Heterogeneity: Not applicable				
Test for overall effect: Z = 2.24 (P = 0.03)				
**1.24.2 Conditional Cash Transfer**				
Macours 2008	0.079	0.04	100.0%	0.08 [0.00, 0.16]
**Subtotal (95% CI)**			**100.0%**	**0.08 [0.00, 0.16]**
Heterogeneity: Not applicable				
Test for overall effect: Z = 1.98 (P = 0.05)				

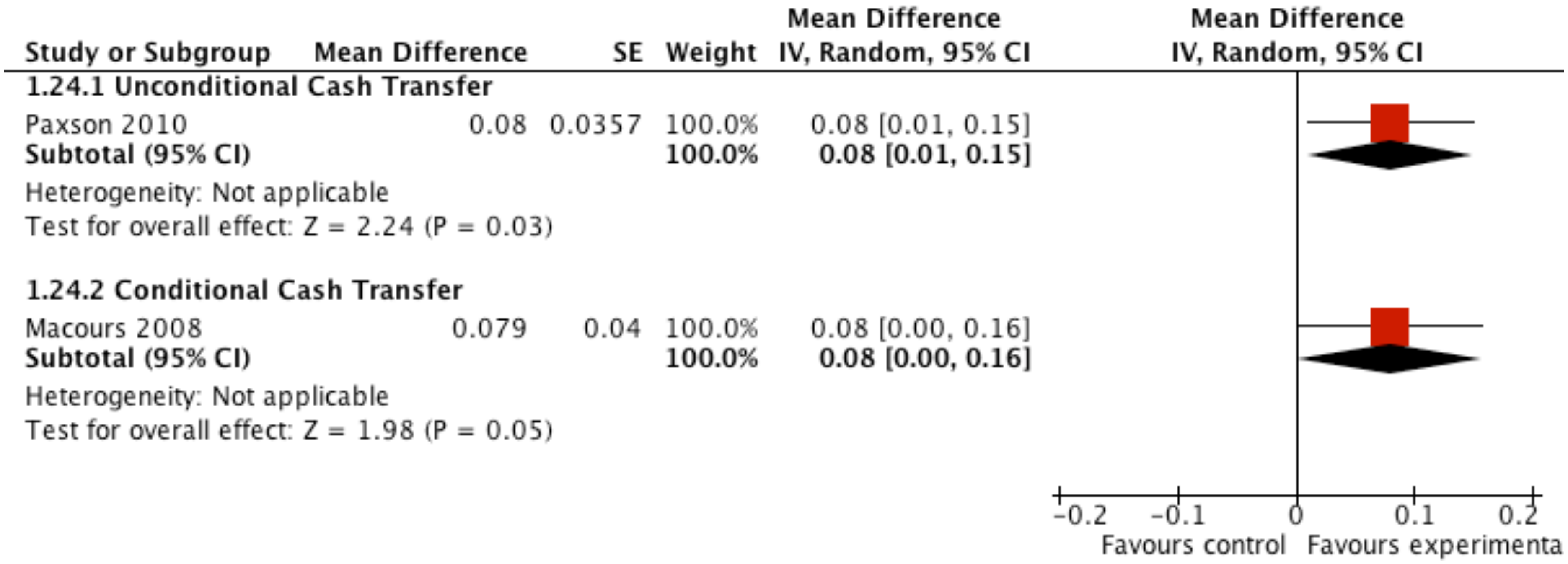

25. Effect of financial incentives on percentage of children receiving vitamin A supplementation in the previous 6 months

Study or Subgroup	Mean Difference	SE	Weight	Mean Difference IV, Random, 95% CI
**1.25.1 Unconditional Cash Transfer**				
Paxson 2010	0.005	0.0179	100.0%	0.01 [−0.03, 0.04]
**Subtotal (95% CI)**			**100.0%**	**0.01 [−0.03, 0.04]**
Heterogeneity: Not applicable				
Test for overall effect: Z = 0.28 (P = 0.78)				
**1.25.2 Conditional Cash Transfer**				
Macours 2008	0.081	0.0117	53.2%	0.08 [0.06, 0.10]
Roy 2008	0.2588	0.0468	46.8%	0.26 [0.17, 0.35]
**Subtotal (95% CI)**			**100.0%**	**0.16 [−0.01, 0.34]**
Heterogeneity: $Tau^2 = 0.01$; $Chi^2 = 13.58$, df = 1 (P = 0.0002); $I^2 = 93\%$				
Test for overall effect: Z = 1.85 (P = 0.06)				

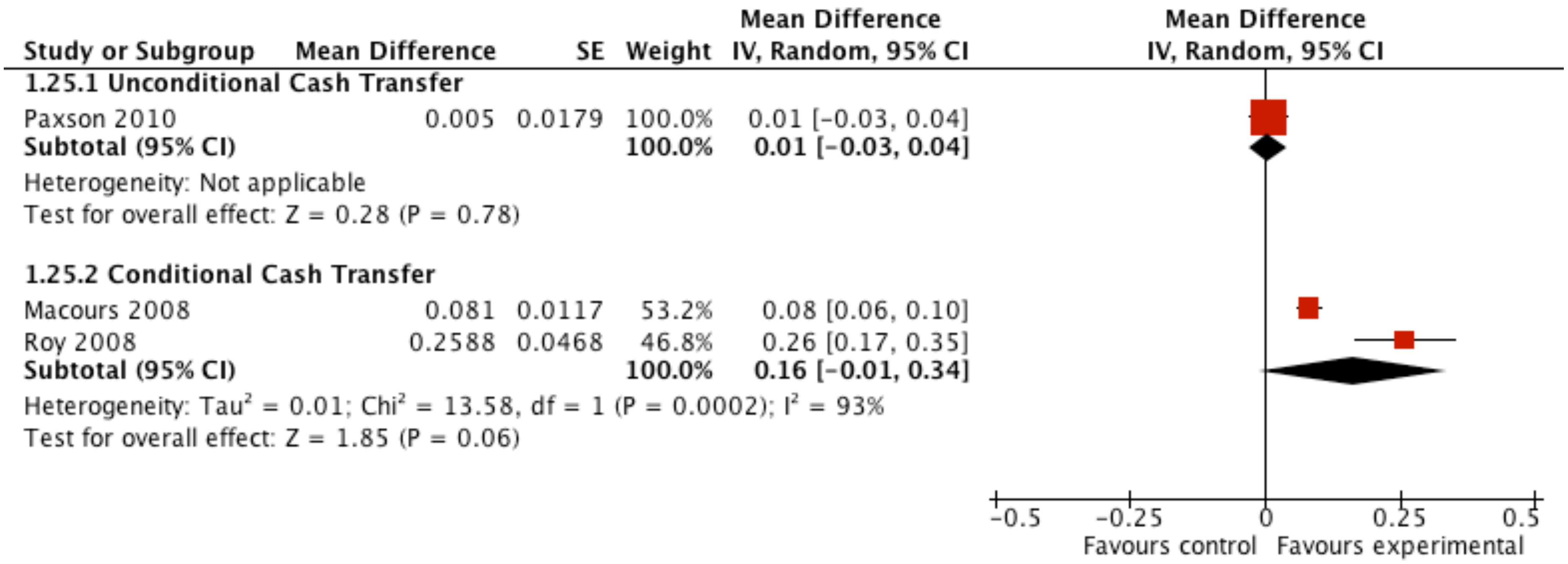

26. Effect of financial incentives on percentage of children receiving iron supplementation in the previous 6 months

Study or Subgroup	Mean Difference	SE	Weight	Mean Difference IV, Random, 95% CI
**1.26.1 Unconditional Cash Transfer**				
Paxson 2010	0.01	0.0204	100.0%	0.01 [−0.03, 0.05]
**Subtotal (95% CI)**			**100.0%**	**0.01 [−0.03, 0.05]**
Heterogeneity: Not applicable				
Test for overall effect: Z = 0.49 (P = 0.62)				
**1.26.2 Conditional Cash Transfer**				
Maluccio 2004	0.361	0.055	100.0%	0.36 [0.25, 0.47]
**Subtotal (95% CI)**			**100.0%**	**0.36 [0.25, 0.47]**
Heterogeneity: Not applicable				
Test for overall effect: Z = 6.56 (P < 0.00001)				

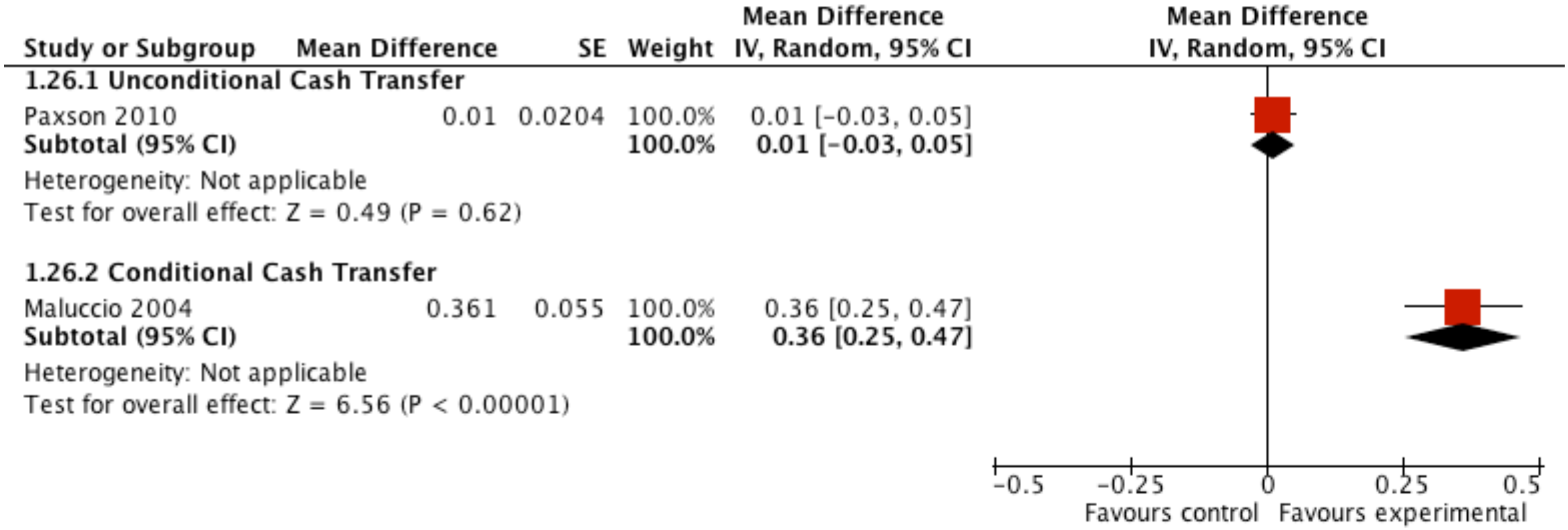

Supplement: Additional File 3 — Forest plots for all outcomes. [file 1471-2458-13-S3-S30-S3.pdf]
